# Phosphoglucomutase is absent in *Trypanosoma brucei* and redundantly substituted by phosphomannomutase and phospho-*N*-acetylglucosamine mutase

**DOI:** 10.1111/j.1365-2958.2012.08124.x

**Published:** 2012-07-12

**Authors:** Giulia Bandini, Karina Mariño, M Lucia Sampaio Güther, Amy K Wernimont, Sabine Kuettel, Wei Qiu, Shamshad Afzal, Anna Kelner, Raymond Hui, Michael A J Ferguson

**Affiliations:** 1 Division of Biological Chemistry and Drug Discovery, College of Life Sciences, University of DundeeDundee DD1 5EH, UK; 2 Structural Genomics Consortium, University of TorontoToronto, Ontario, Canada

## Abstract

The enzymes phosphomannomutase (PMM), phospho-*N*-acetylglucosamine mutase (PAGM) and phosphoglucomutase (PGM) reversibly catalyse the transfer of phosphate between the C6 and C1 hydroxyl groups of mannose, *N*-acetylglucosamine and glucose respectively. Although genes for a candidate PMM and a PAGM enzymes have been found in the *Trypanosoma brucei* genome, there is, surprisingly, no candidate gene for PGM. The *Tb*PMM and *Tb*PAGM genes were cloned and expressed in *Escherichia coli* and the *Tb*PMM enzyme was crystallized and its structure solved at 1.85 Å resolution. Antibodies to the recombinant proteins localized endogenous *Tb*PMM to glycosomes in the bloodstream form of the parasite, while *Tb*PAGM localized to both the cytosol and glycosomes. Both recombinant enzymes were able to interconvert glucose-phosphates, as well as acting on their own definitive substrates. Analysis of sugar nucleotide levels in parasites with *Tb*PMM or *Tb*PAGM knocked down by RNA interference (RNAi) suggests that, *in vivo*, PGM activity is catalysed by both enzymes. This is the first example in any organism of PGM activity being completely replaced in this way and it explains why, uniquely, *T. brucei* has been able to lose its PGM gene. The RNAi data for *Tb*PMM also showed that this is an essential gene for parasite growth.

## Introduction

The kinetoplastid *Trypanosoma brucei* is the causative agent of human African trypanosomiasis, also known as ‘sleeping sickness’, and the cattle disease Nagana. Human African trypanosomiasis is always fatal if untreated and constitutes a major public health problem in sub-Saharan Africa (Favre *et al*., 2008). The disease is characterized by an asymptomatic period of several weeks or months that progresses through an early stage, characterized by malaria-like symptoms, to an advanced stage, where severe neurological and mental disorders appear. There is a clear need for new therapeutics to control the disease.

*Trypanosoma brucei* and the related trypanosomatid parasites *Trypanosoma cruzi* and *Leishmania* sp. synthesize complex cell-surface glycoconjugates, several of which are essential to parasite survival and/or infectivity. Examples for the infective bloodstream form of *T. brucei* include the variant surface glycoprotein (VSG), the transferrin receptor (Steverding *et al*., 1995; Cross, 1996; Mehlert *et al*., 1998; Pays and Nolan, 1998; Mehlert and Ferguson, 2007), the p67 lysosomal glycoprotein (Peck *et al*., 2008) and the membrane-bound histidine acid phosphatase *Tb*MBAP1 (Engstler *et al*., 2005). This has led to the investigation of the enzymes of sugar nucleotide biosynthesis as potential therapeutic targets (Davis *et al*., 2004; Urbaniak *et al*., 2006a, b; Turnock and Ferguson, 2007; Turnock *et al*., 2007; Stokes *et al*., 2008; Denton *et al*., 2010; Lackovic *et al*., 2010; Mariño *et al*., 2010; 2011).

Sugar nucleotides are activated forms of sugars used as donors in glycosylation reactions. They are synthesized either by a *de novo* pathway, requiring the conversion of a precursor sugar/sugar nucleotide, and/or by a salvage pathway, in which the sugar is activated using a kinase and a pyrophosphorylase. In most eukaryotes, sugar nucleotides are formed and used in the cytosol and/or transported in the lumen of the Golgi apparatus and/or the endoplasmic reticulum where they are used by glycosyltransferases (Freeze and Elbein, 2008).

**Fig. 1:**
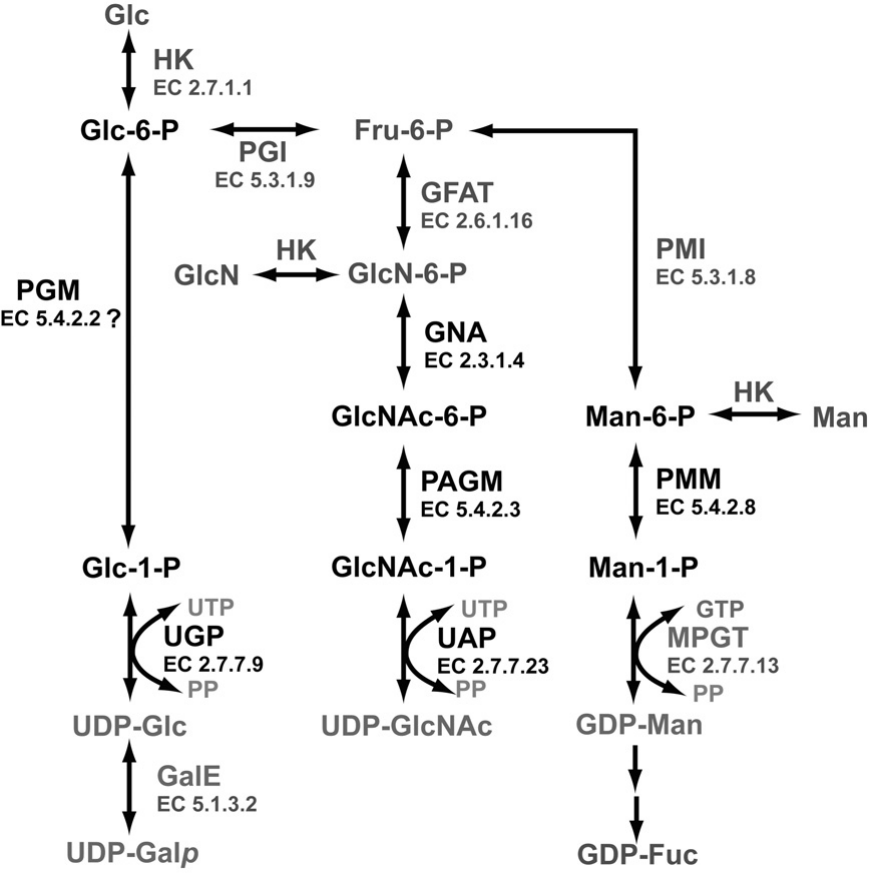
Biosynthesis of UDP-GlcNAc, UDP-Glc, UDP-Gal*p*, GDP-Man and GDP-Fuc in *T. brucei*. Glucose is taken up from the environment and phosphorylated to Glc-6-P, which is the starting point for the *de novo* synthesis of sugar nucleotides. Glc-6-P is normally converted to Glc-1-P by action of a PGM; however, no conventional PGM homologue can be identified in *T. brucei*. Salvage pathways are present for the biosynthesis of GDP-Man and UDP-GlcNAc from Man and GlcN, whereas UDP-Gal*p* can only be synthesized *de novo* from UDP-Glc by action of the UDP-galactose 4-epimerase. The metabolites and enzymes referred to in this paper are marked in *black*. HK, hexokinase; PGI, glucose-6-phosphate isomerase; GFAT, glucosamine-fructose-6-phosphate aminotransferase; GalE, UDP-galactose 4-epimerase; PMI, phosphomannose isomerase; MPGT, GDP-mannose pyrophosphorylase.

In the last few years, our knowledge of *T. brucei* sugar nucleotide biosynthesis (Fig. 1) has widened to demonstrate that several steps in the biosynthesis of GDP-fucose (GDP-Fuc) (Turnock *et al*., 2007), UDP-galactose (UDP-Gal) (Roper *et al*., 2002; 2005; Urbaniak *et al*., 2006b), UDP-*N*-acetylglucosamine (UDP-GlcNAc) (Stokes *et al*., 2008; Mariño *et al*., 2011) and GDP-mannose (GDP-Man) (Denton *et al*., 2010) are essential for parasite growth. Furthermore, most of these, and other (Mariño *et al*., 2010), reports show that many of the enzymes of sugar nucleotide biosynthesis are located in the kinetoplastid peroxisome-like organelle, the glycosome.

Despite these advances, and its importance as a metabolite, relatively little is known about the synthesis of UDP-glucose (UDP-Glc) in *T. brucei*. In eukaryotic cells, UDP-Glc is required for the synthesis of diverse glucose-containing glycoconjugates and secondary metabolites (Flores-Díaz *et al*., 1997). It also plays a crucial role in the ‘quality control’ of newly synthesized glycoproteins taking place in the endoplasmic reticulum (Trombetta and Parodi, 2003; Ruddock and Molinari, 2006), as unfolded glycoprotein glucosyltransferase (UGGT), part of the calnexin- and/or calreticulin-mediated glycoprotein quality control system, requires UDP-Glc as donor substrate (Sousa *et al*., 1992; Caramelo *et al*., 2004). In *T. brucei* UGGT has been shown to be essential for parasite growth and survival at 40°C (Izquierdo *et al*., 2009). UDP-Glc is also the presumed donor for the synthesis of base J (β-D-glucosylhydroxymethyluracil), a rare deoxynucleotide of unknown function found in the DNA of trypanosomatids (Borst and Sabatini, 2008 and references within). Finally, via the action of UDP-Glc 4′-epimerase, UDP-Glc is the only source of the essential sugar nucleotide UDP-Gal in *T. brucei* and *T. cruzi* (Roper *et al*., 2002; 2005; MacRae *et al*., 2006) because their hexose transporters do not take up D-Gal (Barrett *et al*., 1995; Tetaud *et al*., 1996).

Generally, UDP-Glc is synthesized in a three-step process: (i) phosphorylation of glucose to glucose-6-P via hexokinase, (ii) transfer of the phosphate group from C6 to C1 to produce glucose-1-phosphate (Glc-1-P) via phosphoglucomutase (PGM, E.C. 5.4.2.2) and (iii) coupling of glucose-1-P to UTP via UDP-Glc pyrophosphorylase (UGP). In *T. brucei*, the enzymes responsible for the first and last step have been characterized (Nwagwu and Opperdoes, 1982; Mariño *et al*., 2010) and *T. cruzi* and *Leishmania* have easily identifiable PGM genes (Penha *et al*., 2005; 2009). The absence of a putative *T. brucei* PGM gene is therefore perplexing.

The PGMs belong to the α-D-phosphohexomutase (PHM) superfamily of enzymes together with eukaryotic phospho-*N*-acetylglucosamine mutases (PAGM, E.C. 5.4.2.3) and bacterial phosphomannomutase/phosphoglucomutases (PMM/PGM, E.C. 5.4.2.2) and phosphoglucosamine mutases (E.C. 5.4.2.10) (Shackelford *et al*., 2004). Although eukaryotic phosphomannomutases (PMM, E.C. 5.4.2.8) reversibly catalyse the transfer of the phosphate group of a mannosyl phosphate between the C6 and C1 positions, these enzymes belong to the haloacid dehydrogenase superfamily of phosphotransferases (Collet *et al*., 1998).

The apparent lack of a gene encoding for *T. brucei* PGM could be explained if *Tb*PGM bears little or no resemblance to conventional PGMs, or if the conversion of glucose-phosphates can be catalysed by *Tb*PMM and/or *Tb*PAGM. The latter seems the more likely as, although eukaryotic PMMs and PAGMs are generally highly specific for their sugar phosphate substrates, the ability of some PMMs and PAGMs to interconvert glucose-phosphates has been shown (Fernandez-Sorensen and Carlson, 1971; Boles *et al*., 1994; Hofmann *et al*., 1994; Oesterhelt *et al*., 1996; Pirard *et al*., 1997; 1999; Kato *et al*., 2005; Qian *et al*., 2007).

In this paper, we identified the genes encoding functional *T. brucei* phosphomannomutase (*Tb*PMM) and phospho-*N*-acetylglucosamine mutase (*Tb*PAGM) and studied their subcellular location and metabolic activities in bloodstream form *T. brucei*. As well as interconverting mannose- and *N*-acetylglucosamine-phosphates respectively, both were shown to be capable of producing Glc-1-P from glucose-6-phosphate (Glc-6-P), albeit with different efficiencies. *Tb*PMM was also crystallized and its structure solved at high resolution. The dual specificities of these enzymes suggested an explanation for the absence of a *Tb*PGM gene in this parasite and knockdown of *Tb*PMM and *Tb*PAGM by RNA interference (RNAi) showed that both enzymes are mutually redundant for their PGM activity.

## Results

### Identification of *T. brucei* PMM and PAGM encoding genes

**Fig. 2:**
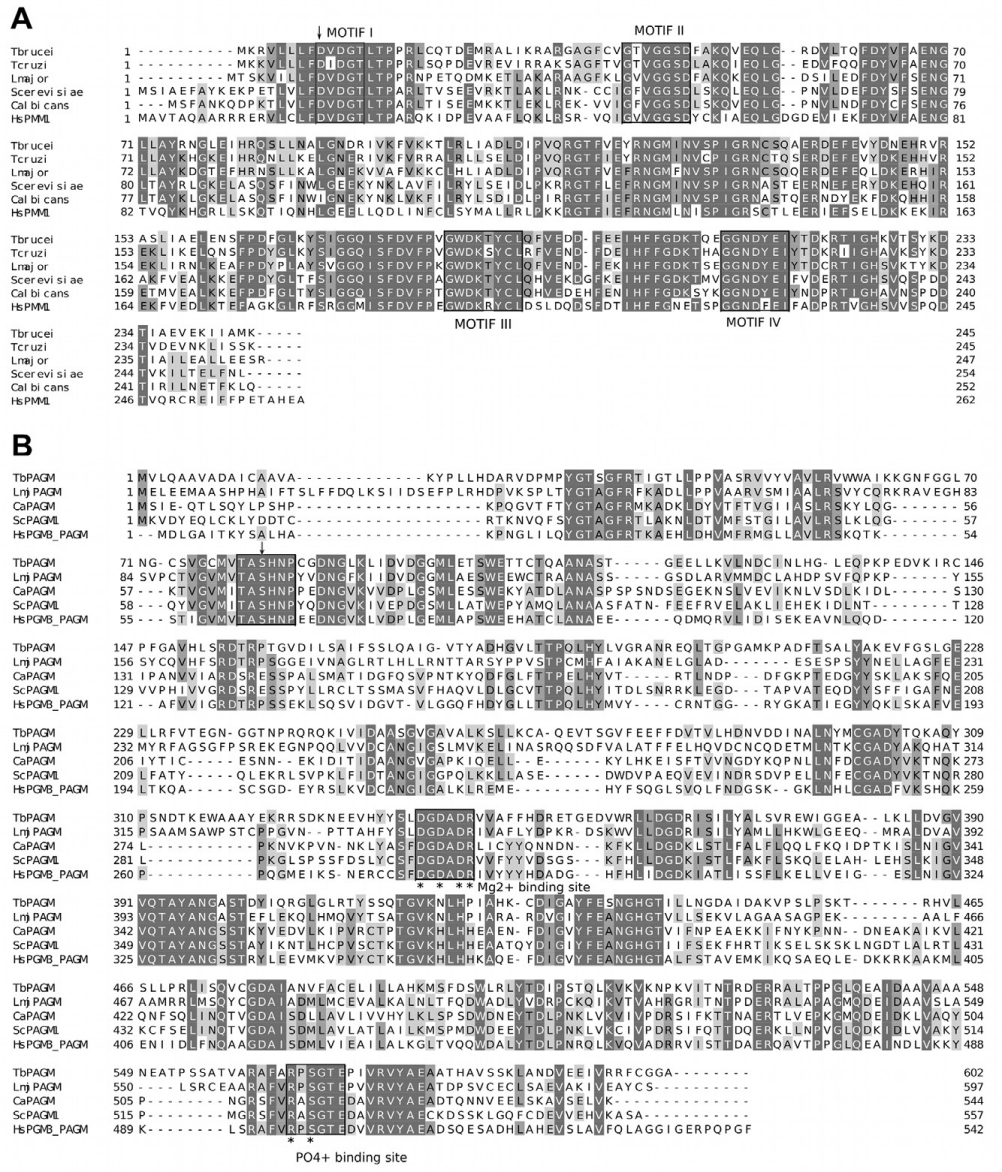
*Tb*PMM and *Tb*PAGM show homology to their eukaryotic orthologues. Amino acids conserved between all proteins are highlighted in *dark grey*. Sequences from protozoa (*T. brucei, T. cruzi* and *L. major*), yeasts (*S. cerevisiae* and *Candida albicans*) and mammals (*Homo sapiens* PMM1) were used for the alignments. A. Sequences from the eukaryotic PMM family. The four conserved motifs are indicated by *black boxes*, while the *arrow* points to the Asp residue that is phosphorylated during the reaction. B. PAGM sequence alignment. Conserved motifs for the α-D-PHM family are indicated by *black boxes*, the *asterisks* indicate invariant residues and the *arrow* points to the P-Ser residue.

*Saccharomyces cerevisiae* PMM (NCBI accession no.: NP116609) was previously used as a template for a BLASTp search of *T. brucei* predicted proteins and **Tb10.70.0370** was identified as a putative *TbPMM*. Putative PMM genes could be also found in the *Leishmania major* (GeneDB ID: **LmjF36.1960**) and *T. cruzi* (GeneDB ID: **TcCLB.510187.480**) genomes (Turnock and Ferguson, 2007) and the *Leishmania mexicana* PMM has been structurally characterized (**LmxM.36.1960**; Kedzierski *et al*., 2006). As shown in Fig. 2A, *Tb*PMM belongs to the eukaryotic PMM family. Eukaryotic PMMs are members of the haloacid dehydrogenase superfamily of phosphotransferases. *Tb*PMM, like the other eukaryotic PMMs, belongs to the type II class of this superfamily (Kedzierski *et al*., 2006; Silvaggi *et al*., 2006). This can be deduced by the location of the cap domain (aa 85–183), which in this class is found between motif II and III (Fig. 2A). Eukaryotic PMMs are characterized by a conserved DVDGT motif (motif I, aa 9–13), localized at the protein N-terminus. The first Asp of this motif (D9) is phosphorylated during the catalytic cycle. *Tb*PMM shows 57% sequence identity to its *S. cerevisiae* homologue.

A BLASTp search of the *T. brucei* predicted protein database with *Homo sapiens* and *S. cerevisiae* PAGM amino acid sequences (NCBI accession nos. NP_056414.1 and NP_010856.1 respectively) revealed a single putative *TbPAGM* gene (GeneDB ID: **Tb927.8.980**) (Turnock and Ferguson, 2007). Two putative PAGMs could also be identified in *T. cruzi* (GeneDB ID: **TcCLB.503733.70**, **TcCLB.508569.80**). Both genes are more than 97% identical, probably accounting for the two haplotypes (Esmeraldo and non-Esmeraldo-like) present in the CL-Brener strain that was sequenced (El-Sayed *et al*., 2005a). A single PAGM gene was also detected in *L. major* (GeneDB ID: **LmjF07.0805**). For *Tb*PAGM, the predicted amino acid sequence contains the conserved motifs for the α-D-PHM family, such as the phosphate-binding site (RPSGTE, aa 564–569), the Mg^2+^ binding site (DGDADR, aa 338–343), and also several invariant residues such as R564, S566, G418, D338, D340, D342, R343 (marked by asterisks, Fig. 2B) (Shackelford *et al*., 2004). However, in *Tb*PAGM, the sugar-binding site (aa 435–442) contains an unusual variation, whereby the normally highly conserved A437 residue is replaced by a serine. As observed in other PAGMs, the phosphoserine loop (TASHNP, aa 80–85, black box, Fig. 2B), essential for catalytic activity, is translocated to an earlier position compared to other α-PHM family members, in order to accommodate the bulky *N*-acetyl group present in the phosphorylated GlcNAc substrate (Shackelford *et al*., 2004). *Tb*PAGM shares 28% and 35% identity to its human and yeast orthologues respectively.

According to a recent quantitative proteomic analysis, both *Tb*PAGM and *Tb*PMM are present at the protein level in both life cycle stages and the relative concentrations of the proteins (procyclic levels divided by bloodstream form levels) are 1.06 ± 0.30 and 1.17 ± 0.30 respectively (Urbaniak *et al*., 2012).

### The activities of recombinant *Tb*PMM and *Tb*PAGM on mannose- and N-acetylglucosamine-phosphates

The *TbPMM* and *TbPAGM* open reading frames (ORFs) were amplified by PCR from genomic DNA obtained from *T. brucei* strain 427, sequenced (EMBL nucleotide sequence database accession number FR851306 and FR851307) and cloned into an expression vector containing *N*-terminal His_6_-tags and expressed in *E. coli*. After Nickel column purification, *Tb*PMM protein was further purified by size exclusion chromatography (Fig. S1) and the His-tag was removed by incubation with TEV protease resulting in a band of 27.9 kDa by SDS-PAGE (Fig. S1). For *Tb*PAGM, the His-tag was cleaved with PreScission protease™ and the untagged protein was further purified by size exclusion chromatography (Fig. S2). The molecular masses and identities of the tagged and untagged recombinant proteins were verified by MALDI-TOF and by LC-MS/MS peptide mass fingerprinting respectively (data not shown). Although both recombinant enzymes were active with and without the His-tag, the untagged recombinant enzymes were used for activity assays and, in the case of *Tb*PMM, for crystallization trials.

**Fig. 3:**
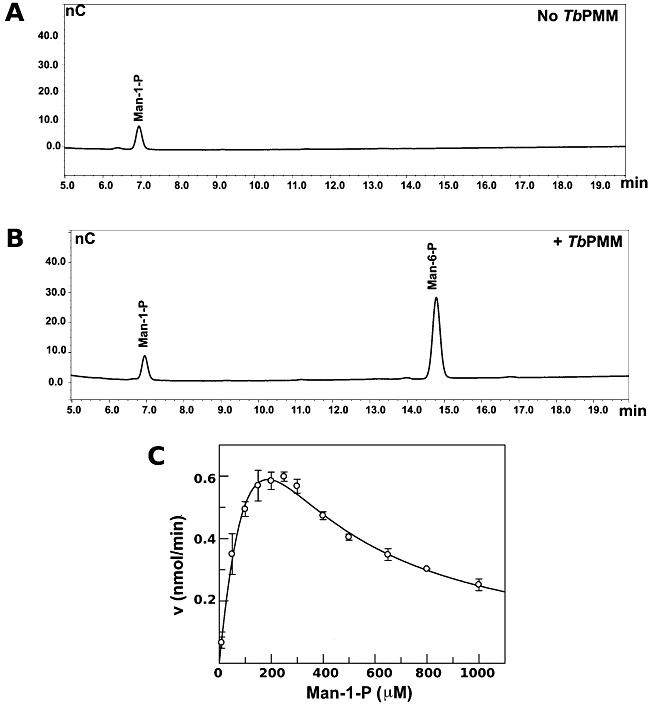
*Tb*PMM converts Man-1-P to Man-6-P. Recombinant *Tb*PMM was incubated with Man-1-P and the Glc-1,6-biphosphate cofactor and the products of the reaction were analysed by HPAEC-PAD. A peak corresponding to Man-6-P was observed in the presence of the recombinant enzyme (B), but not in its absence (A). C. Kinetic analysis of *Tb*PMM which has an apparent *K_m_* for Man-1-P of 327 ± 66 µM and a maximum velocity of 2.6 ± 0.4 nmol min^−1^ per milligram of protein (Table 1).

A high-pH anion exchange chromatography with pulsed amperometric detection (HPAEC-PAD) system was used to assay the activity of the recombinant *Tb*PMM, as described in *Experimental procedures*. It was clear that *Tb*PMM can convert Man-1-P to Man-6-P in the presence of glucose-1,6-biphosphate (Glc-1,6-biP) as a cofactor (Fig. 3B), whereas no Man-6-P could be detected when *Tb*PMM was absent (Fig. 3A). We used the same system to see whether *Tb*PMM could convert GlcNAc-1-P to GlcNAc-6-P, but this was not the case (Fig. S3A). The same procedure was also used to determine the kinetic properties of the enzyme for Man-1-P (Fig. 3C). Different concentrations of Glc-1,6-biP were tested before selecting an optimal concentration of 1 µM. The apparent *K_m_, V_max_* and catalytic efficiency of recombinant *Tb*PMM using Man-1-P as substrate are shown in Table 1. It was not possible to saturate the reaction, as the *Tb*PMM activity showed inhibition at the highest Man-1-P concentrations (Fig. 3C). Consequently, an equation for high-substrate inhibition was used to calculate the kinetic parameters of the reaction. The pH dependence of *Tb*PMM PMM activity was analysed over the pH range 4.0 to 10.0. The enzyme showed activity between pH 5.0 and 8.0 with an optimum at pH 6.5 (data not shown).

**Table 1:** Comparison of the kinetic properties of recombinant *Tb*PMM and *Tb*PAGM with different sugar phosphates as substrates

	*Tb*PMM			*Tb*PAGM				
		
							IC_50_ (µM)^a^	
							
Substrate	K_m_ (µM)	v_max_ (nmol min^−1^)	K_cat_/K_m_ (M^−1^ s^−1^)	K_m_ (µM)	v_max_ (nmol min^−1^)	K_cat_/K_m_ (M^−1^ s^−1^)	GlcNAc-6-P	GlcNAc-1-P
Man-1-P	327 ± 66^b^	2.6 ± 0.4	3.7 × 10^5^					
Glc-6-P	96 ± 8^c^	0.331 ± 0.008	5.7 × 10^4^	107 ± 17^b^	0.23 ± 0.03	9.4 × 10^4^	8.3 ± 0.4	6.3 ± 2.6
GlcNAc-6-P				14 ± 6^c^	0.051 ± 0.006	1.6 × 10^5^		

a. IC_50_ for GlcNAc phosphates as competitive inhibitors in the formation of Glc-1-P.

b. The kinetic constants were calculated using an equation for high-substrate inhibition based on the best-fit non-linear lines.

c. The kinetic constants were calculated from the Michaelis–Menten equation.

**Fig. 4:**
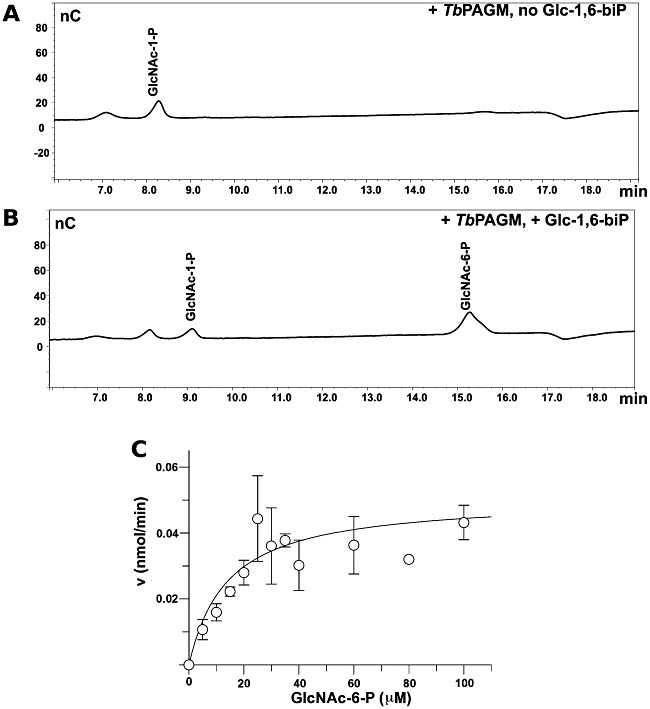
*Tb*PAGM interconverts GlcNAc phosphates. Recombinant *Tb*PAGM was incubated with GlcNAc-1-P with and without the Glc-1,6-biphosphate cofactor, as indicated, and the products of the reaction were analysed by HPAEC-PAD. A peak corresponding to GlcNAc-6-P was observed in the presence of the recombinant enzyme and cofactor (B), but not when the cofactor was removed from the reaction (A). C. Kinetic analysis of *Tb*PMM which has an apparent *K_m_* for GlcNAc-6-P of 14 ± 6 µM and a maximum velocity of 0.051 ± 0.006 nmol min^−1^ per milligram of protein (Table 1).

The HPAEC-PAD system was also used to show the activity of recombinant *Tb*PAGM on GlcNAc-1-P and GlcNAc-6-P, with Glc-1,6-biP as cofactor (Fig. 4B). *Tb*PAGM was also able to convert Man-1-P to Man-6-P *in vitro* (Fig. S3B); however, as discussed later, this activity was not able to compensate for the knockdown of *Tb*PMM *in vivo*. The kinetic parameters for the conversion of GlcNAc-6-P to GlcNAc-1-P by *Tb*PAGM were determined using a *Tb*UAP and pyrophosphorylase coupled assay with colorimetric detection (Stokes *et al*., 2008) in a 96-well plate format, as described under *Experimental procedures*. The *Tb*PAGM enzyme followed classic Michaelis–Menten kinetics (Fig. 4C) and the apparent *K_m_, V_max_* and catalytic efficiency of recombinant *Tb*PMM with using GlcNAc-6-P as the substrate are shown in Table 1. The pH dependence of *Tb*PAGM was studied over a 4.0 to 10.0 pH range. The enzyme showed a broad optimum between pH 7.2 and 9.1 (data not shown).

### Both *Tb*PMM and *Tb*PAGM can convert Glc-6-P to Glc-1-P

**Fig. 5:**
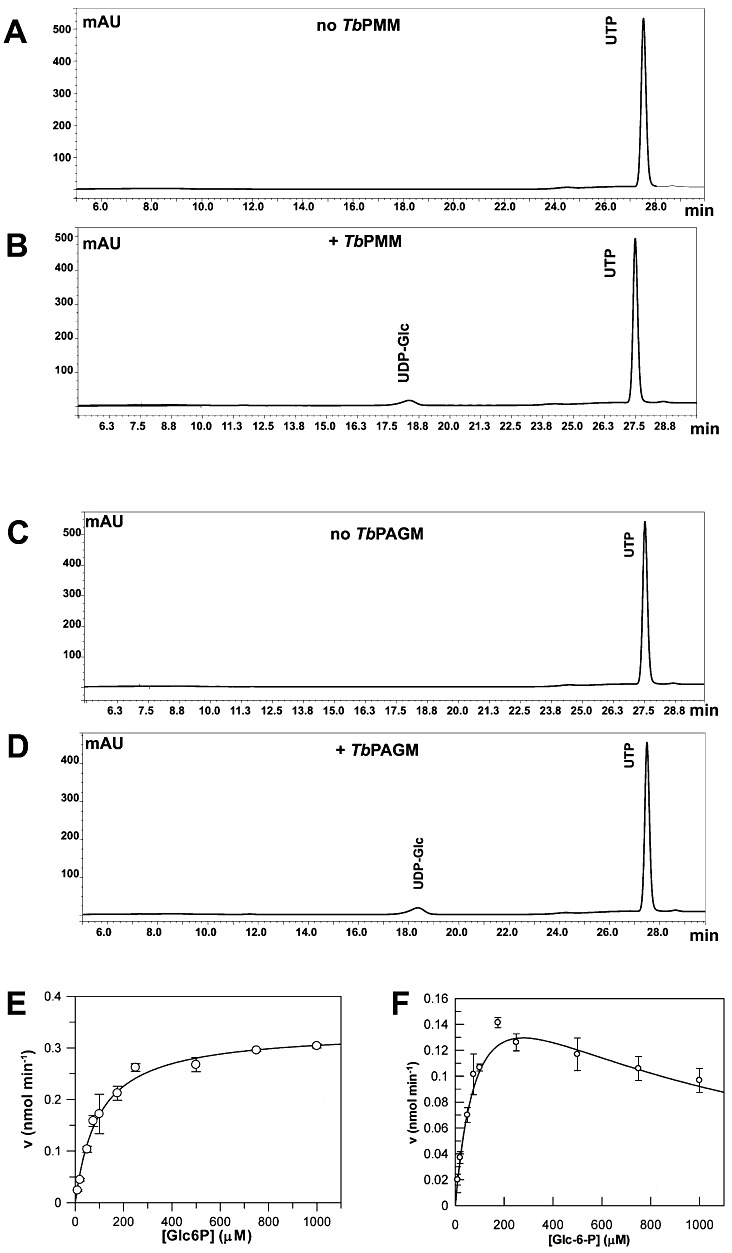
*Tb*PMM an*d Tb*PAGM can convert Glc-6-P to Glc-1-P*. Tb*PMM and *Tb*PAGM were used in a coupled assay with *Tb*UGP in the presence of Glc-6-P and UTP, as indicated. The products of the reactions were analysed by HPAEC with UV detection. Formation of UDP-Glc (indicative of the conversion of Glc-6-P to Glc-1-P) was observed with both *Tb*PMM (B) and *Tb*PAGM (D). No sugar nucleotide peak could be detected in the absence of either phospho-sugar mutase (A and C). The kinetic analyses of recombinant *Tb*PMM (E) and *Tb*PAGM (F) were performed using the *Tb*UGP-coupled assay with colorimetric detection. The results are in Table 1.

The ability of *Tb*PMM and *Tb*PAGM to catalyse this reaction was assessed by incubating each recombinant enzyme in the presence of Glc-1-P. In both cases the formation of Glc-6-P could be detected by HPAEC-PAD (Fig. S4). As Glc-1-P is the substrate for the UGP, we were able to set up an assay for the conversion of Glc-6-P to Glc-1-P by coupling *Tb*PMM and *Tb*PAGM with *Tb*UGP to produce UDP-Glc in the presence of UTP (Mariño *et al*., 2010). Thus, a single peak that co-eluted with UDP-Glc could be detected on a HPAEC-UV system at A_260_, when either *Tb*PMM or *Tb*PAGM was coupled with recombinant *Tb*UGP (Fig. 5B and D). Removal of either PHM from the reaction resulted in the loss of the UDP-Glc peak (Fig. 5A and C).

The kinetic parameters for the conversion of Glc-6-P to Glc-1-P by *Tb*PMM and *Tb*PAGM were determined in a discontinuous coupled assay with *Tb*UGP with colorimetric detection. This assay measures the pyrophosphate (PPi) product of the Glc-1-P + UTP → UDP-Glc + PPi coupling reaction via conversion of the PPi to inorganic phosphate and detection with a molybdate reagent (see *Experimental procedures*). The kinetic values of *Tb*PMM and *Tb*PAGM are listed in Table 1. Interestingly, *Tb*PAGM activity was inhibited at the highest concentrations of Glc-6-P so that no saturation could be observed in the kinetic analysis (Fig. 5F). No such inhibition could be detected in the case of *Tb*PMM (Fig. 5E).

**Fig. 6:**
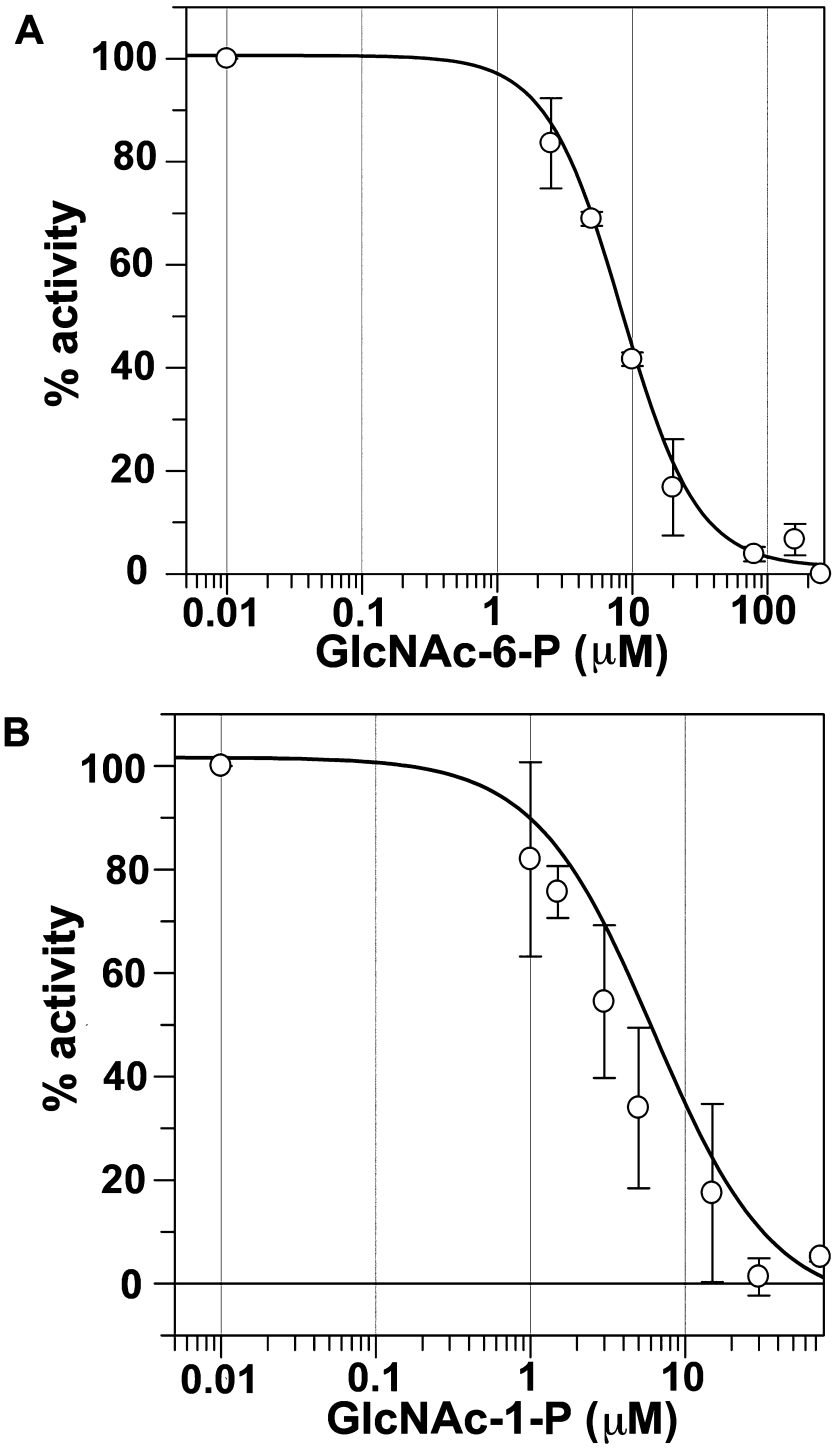
GlcNAc-6-P and GlcNAc-1-P can inhibit the formation of UDP-Glc *in vitro*. When *Tb*PAGM was used as the source of PGM activity in the first step of UDP-Glc formation, the biosynthesis of this sugar nucleotide could be inhibited by the presence of both GlcNAc-6-P (A) and GlcNAc-1-P (B). Thus, both sugar phosphates compete with Glc-6-P for binding to *Tb*PAGM with IC_50_ values of 8.3 ± 0.4 µM and 6.3 ± 2.6 µM respectively (Table 1).

As the affinity of *Tb*PAGM for GlcNAc-6-P is almost 10-fold higher than for Glc-6-P, we investigated whether GlcNAc-6-P could act as a competitive inhibitor in the formation of Glc-1-P. This was confirmed by running the assay at 100 µM Glc-6-P (i.e. at the apparent K_m_ of *Tb*PAGM for Glc-6-P) in the presence of increasing concentrations of GlcNAc-6-P, which had an IC_50_ of 8.3 ± 0.4 µM (Fig. 6A). GlcNAc-1-P also inhibited the conversion of Glc-6-P to Glc-1-P with a comparable IC_50_ of 6.3 ± 2.6 µM (Fig. 6B).

### Determination and analysis of the *Tb*PMM structure

The crystal structure of the full-length *Tb*PMM was solved (PDB accession number **3F9R**) by molecular replacement using a modified form of the *L. mexicana* PMM (*Lm*PMM; PDB accession number **2i54**). The crystallographic and model refinement data for the *Tb*PMM structure are reported in Table 2. Two polypeptide units could be seen in the crystals asymmetric unit, chains A and B. When symmetry mates were extended, each monomer formed a dimer with its own symmetry mates, i.e. A : A and B : B. These dimers mimicked the dimerization mode seen in both human and *Lm*PMM structures (Fig. S6).

**Table 2:** Data collection and crystallographic refinement

Data collection	
Space group	P21212
Cell dimensions	
a (Å)	105.93
b (Å)	46.99
c (Å)	94.49
Wavelength	1.5418
Resolution	50–1.8 (1.86)
Unique reflections	44 877
R_merge_	0.122 (0.862)
Mean I/σ(I)	17.83 (1.40)
Completeness (%)	99.9 (99.3)
Redundancy	6.2 (4.4)
PDB code	3F9R
Refinement	
Resolution	50–1.85
Number of reflections	41 076
Test set	2074
R_work_/R_free_	0.202/0.266
RMS deviations	
Bond lengths (Å)	0.016
Bond angles (°)	1.471
Average B value (Å^2^)	31.63

The oligomeric state of *Tb*PMM was studied by analytical ultracentrifugation (AUC) and size exclusion chromatography. Recombinant *Tb*PMM was analysed by AUC in two different buffers: one containing sodium chloride at physiological concentrations (10 mM HEPES pH 7.5, 150 mM NaCl) and one without any salt (10 mM Tris-HCl pH 7.5, 1 mM DTT). This last buffer is the same used in the crystallization of *Lm*PMM (Kedzierski *et al*., 2006). In both cases, *Tb*PMM behaved as a monomer and no oligomers could be observed (Fig. S5). It should be noted that when *Tb*PMM was analysed by size exclusion chromatography in high salt during the purification process, the retention time was consistent with the apparent molecular weight (MW) of a homodimer (data not shown). Because the dimeric state of *Tb*PMM could be observed only at high concentrations of salt, but not under physiological conditions, we concluded the biological assembly of *Tb*PMM to be a monomer.

**Fig. 7:**
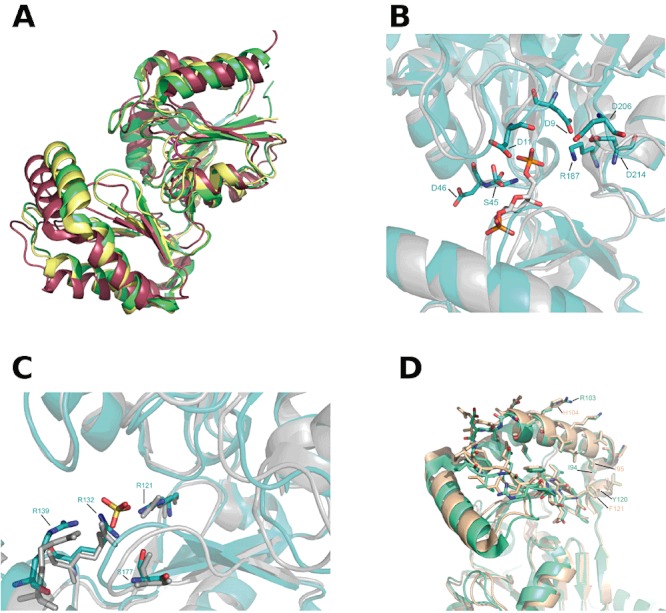
High-resolution crystal structure of *Tb*PMM. A. Comparison of the *Tb*PMM structure (*green*) with monomers from *Lm*PMM (*yellow*) and *H. sapiens* PMM1 (*magenta*). B. Active sites from *Tb*PMM (*blue*) with the Glc-1,6-biP cofactor modelled in compared to *Lm*PMM (*grey*). All four Asp residues that should be involved in the phosphoprotein intermediate (D9) and the Mg^2+^ co-ordination (D11, D206 and D214) are conserved, as are S45 and R187, which are involved in phosphate moiety recognition. S45 is shown in both its possible conformations. C. A sulphate ion was found in a position where the phosphate group from the substrate would be expected. The ion is co-ordinated by R139 and R132 directly and by R121 and S177 through water molecules. *Tb*PMM structure is shown in *blue* and *Lm*PMM in *grey*. D. Comparison of the surface interfaces between *Tb*PMM (*green*) and *Lm*PMM (*beige*). The three mutated residues are labelled.

Comparison of the *Tb*PMM crystal structure with of the monomers of the *L. mexicana* and human enzymes showed that the overall structure is very well conserved (Fig. 7A), as are the residues forming the active site (Fig. 7B). As previously described, the residues involved in the catalysis are located in the core domain (aa 5–82 and 187–246): D9 (Motif I) corresponds to the Asp that is phosphorylated in the reaction, S45 (motif II) and K187 (Motif III) are involved in the binding of the phosphate, and D9, D11 (Motif I), D206 and D214 (Motif IV) co-ordinate the Mg^2+^ ion (Fig. 7B and C) (Allen and Dunaway-Mariano, 2004; Quental *et al*., 2010). The cap domain contributes the residues involved in substrate recognition (E119, R121, M124, N126, R132, R139, S177), so that the active site is located in the groove at the interface of the core and cap domains (Fig. 7A) (Kedzierski *et al*., 2006; Silvaggi *et al*., 2006). The electron density map for the solved structure revealed a sulphate ion in each monomer's active site, in approximately the same position in which the 1-phosphate moiety of the Glc-1,6-biP cofactor would be found (Kedzierski *et al*., 2006). This ion co-ordinates directly with R139 and R132, and through linking waters with S177 and R121 (Fig. 7C). Overlaying the structure with **2i55**, the *Lm*PMM structure with Glc-1,6-biP bound, the phospho-sugar fits quite well within the closed pocket in *Tb*PMM. In Fig. 7B, we can see the strict conservation within this pocket. In the *Lm*PMM structure, D10, D207 and D215 are engaged in co-ordinating a Mg^2+^ ion and D10 is supposed to form the phosphoprotein intermediate and in the *Tb*PMM structure, all four Asp residues are conserved (D9, D11, D206 and D214), even though no Mg^2+^ ion was found in the electron density. No additional magnesium was added to the protein prior to crystallization, although density for a magnesium ion was found in a distal part of the enzyme at another magnesium-binding site found in other PMMs structures (**2i55**, **2fue**).

As the quaternary structure of *Tb*PMM is the main difference between this enzyme and other described eukaryotic PMMs, which are all homodimers (Kepes and Schekman, 1988; Kedzierski *et al*., 2006; Silvaggi *et al*., 2006), we analysed the residues at the hypothetical dimer interface. Analysis using PISA-EBI identified 12 residues potentially involved in H-bonding or salt bridge interactions (N91, K99, R103, A106, D107, D109, P111, R114, T116, V118, Y120 and N133) and 14 residues involved in hydrophobic or other interactions in the *Tb*PMM structure. These residues are well conserved in the *Lm*PMM interface, with the exception of three amino acids. Two of these (I94 and Y120) were partially involved in hydrophobic interactions and correspond to the *Lm*PMM residues V95 and F121. The third substitution occurs at R103, which in *Lm*PMM is H104 (Fig. 7D). Interestingly, the PISA-EBI macromolecular analysis tool finds a low complexation significance score (CSS) for *Tb*PMM (0.017) further suggesting a monomeric quaternary structure. As a comparison, the CSS for *Lm*PMM is 0.249, consistent with the interface residues having a role in multimer formation.

### Subcellular localization of *Tb*PMM and *Tb*PAGM in bloodstream form *T. brucei*

**Fig. 8:**
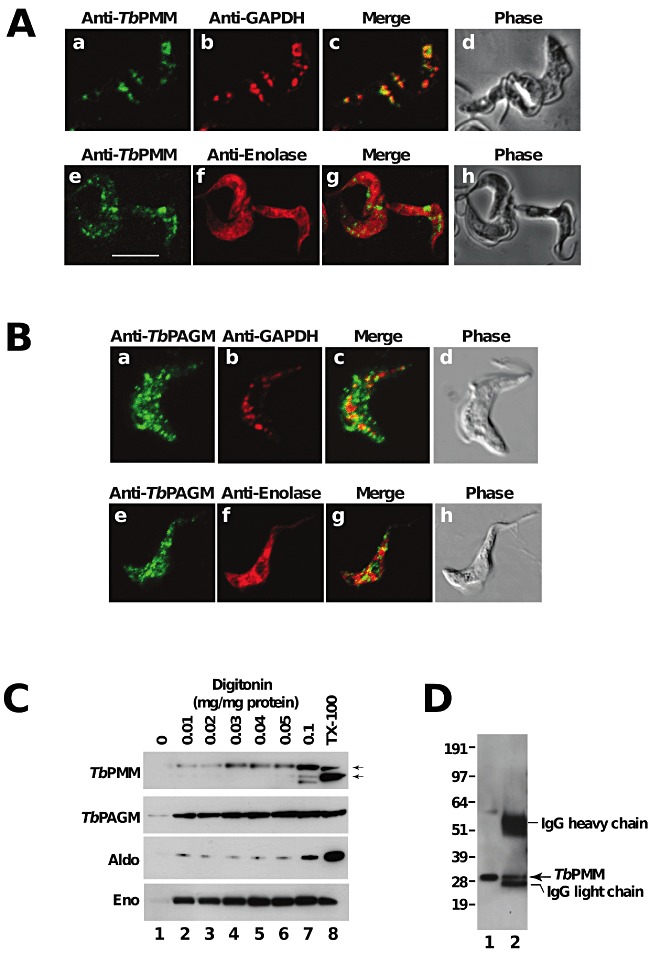
Subcellular localization of *Tb*PMM and *Tb*PAGM. Immunofluorescence microscopy was performed on paraformaldehyde-fixed bloodstream form *T. brucei* cells stained with mouse anti-*Tb*PMM (A) or mouse anti-*Tb*PAGM (B) sera combined with glycosomal marker rabbit anti-GAPDH (images a to d) or cytosolic marker rabbit anti-enolase (images e to h). Merged images are shown in images c and g and corresponding phase contrast images are shown in images d and h. A scale bar (10 µm) is shown in A, image e. The subcellular localization of *Tb*PMM and *Tb*PAGM was also studied by digitonin latency, i.e. the release of specific proteins, as judged by Western blotting with antibodies to *Tb*PMM and *Tb*PAGM, the glycosomal marker aldolase (aldo) and the cytosolic marker enolase (eno), against increasing concentrations of digitonin (C). In the *Tb*PMM blot, the top arrow indicates intact *Tb*PMM while the bottom arrow indicates *Tb*PMM proteolytic products. The specificity of *Tb*PMM antibody (D) was tested by Western blotting of an immunoprecipitate using the same antibody from 2 × 10^8^ bloodstream form cells (lane 2) and also against 25 ng of recombinant *Tb*PMM (lane 1). The MW markers are shown of the left.

Subcellular localization for both enzymes was studied by immunofluorescence microscopy. Paraformaldehyde-fixed wild type bloodstream form *T. brucei* cells were incubated with polyclonal mouse anti-*Tb*PMM (Fig. 8A) or anti-*Tb*PAGM (Fig. 8B) combined either with rabbit polyclonal anti-GAPDH, as glycosomal marker (images a to d) or with rabbit anti-enolase, as cytosolic marker (images e to h). Cells were washed and incubated with a mix of Alexafluor 488 anti-mouse IgG (green) and Alexafluor 594 anti-rabbit IgG (red). Anti-*Tb*PMM showed a punctate staining pattern and substantial colocalization with anti-GAPDH (Fig. 8A, images a to d), and an absence of colocalization with anti-enolase (Fig. 8A, images e to h), indicating that *Tb*PMM is a glycosomal enzyme. On the other hand, *Tb*PAGM showed both punctate and diffuse staining, in agreement with partial colocalization with GAPDH (Fig. 8B, images a to d) and with enolase (Fig. 8B, images e to h), respectively, indicating that *Tb*PAGM is located in both the glycosomes and the cytosol.

The subcellular localization of *Tb*PMM and *Tb*PAGM was also studied by digitonin latency, as described previously (Galland *et al*., 2007), using Western blotting of the proteins released by escalating digitonin concentrations (Fig. 8C). *Tb*PMM was very poorly released at lower concentrations of digitonin but increased gradually with increasing digitonin concentration, reaching a maximum at 0.1 mg digitonin/mg protein. This pattern is very similar to that for aldolase (aldo), a lumenal glycosome resident enzyme, indicating that *Tb*PMM is also a glycosomal enzyme. Lower MW bands were observed at higher concentrations of digitonin and at 0.1% Triton X-100 (Fig. 8C, lanes 7 and 8 respectively) are probably due to proteolytic degradation of *Tb*PMM, and not due to non-specificity of the affinity-purified antibody that specifically immunoprecipitated a single band of the correct apparent MW from 2 × 10^8^ cell equivalents of a trypanosome detergent lysate (Fig. 8D, lane 2).

Digitonin latency was also used to study the cellular location of *Tb*PAGM. Unlike *Tb*PMM, *Tb*PAGM was substantially released at low concentrations of digitonin, a pattern similar to enolase (eno), a cytosolic protein. However, the release of *Tb*PAGM increased gradually with the increase in digitonin concentration (Fig. 8A, compare lanes 2 and 7) indicating that PAGM has a dual localization in the cell, glycosomal and cytosolic.

### RNAi knockdown of *Tb*PMM and *Tb*PAGM and the effects on sugar nucleotide metabolism in bloodstream form *T. brucei*

The contributions of *Tb*PMM and *Tb*PAGM to the biosynthesis of UDP-Glc and UDP-Gal*p* were studied by RNAi. Fragments of 464 and 497 bp of *TbPMM* and *TbPAGM* ORFs, respectively, were cloned into p2T7TA^Blue^ and the resulting constructs were used to generate two cell lines expressing tetracycline inducible double-stranded RNA (dsRNA) targeting *TbPMM* and *TbPAGM* respectively.

**Fig. 9:**
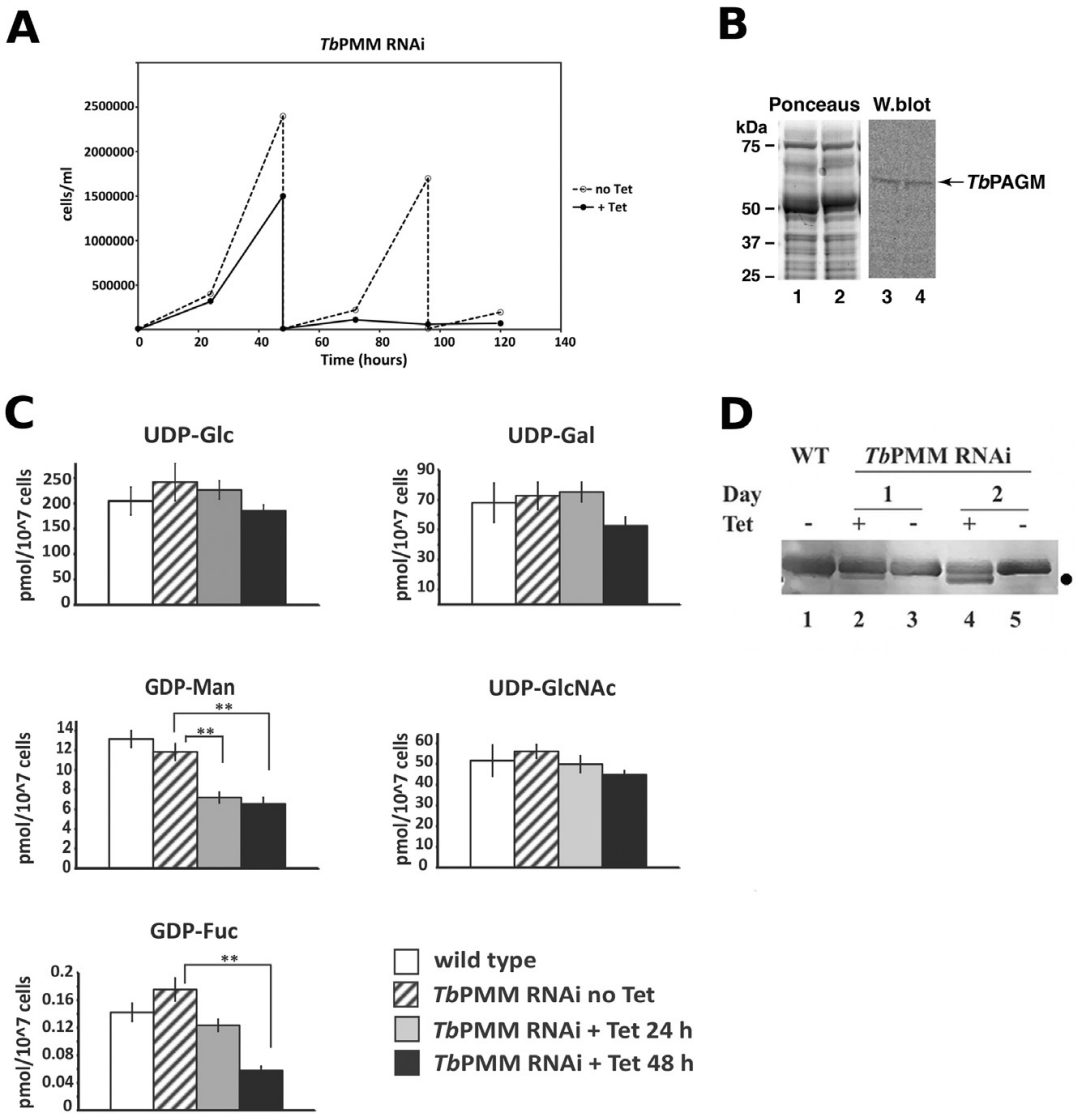
Characterization of *Tb*PMM RNAi effects. A. Growth curves of the *Tb*PMM RNAi cell line induced with tetracycline (*solid line*) or un-induced (*dashed line*). B. Western blot of total cell lysates from un-induced (lanes 1 and 3) or induced (lanes 2 and 4) *Tb*PMM RNAi cells stained with Ponceau (lanes 1 and 2) and with antibodies to *Tb*PAGM (lanes 3 and 4). C. The levels of sugar nucleotide were determined by LC-MS/MS in wild type cells (*white*), the un-induced *Tb*PMM RNAi cell line (*striped*) or the *Tb*PMM RNAi cell line induced for 24 h (*grey*) or 48 h (*black*). The *P*-values (*P*) were determined by *t*-test; the double asterisk indicates a *P* < 0.005. D. The extent of *N*-glycosylation of sVSG221 was assessed by anti-sVSG221 Western blot of whole cell lysates from wild type (WT) cells (lane 1) and *Tb*PMM RNAi cells that were induced (+) or un-induced (−) with tetracycline for 1 day or 2 days, as indicated. The lower band in lanes 2 and 4, marked with a spot, is consistent with sVSG221 lacking one of its two *N*-glycans.

Induction of dsRNA targeting *TbPMM* resulted in a 68 ± 18% knockdown of *Tb*PMM mRNA after 48 h (as judged by qRT-PCR with four replicates) and a reduction of growth rate that led to cell death after 72 h, demonstrating the essentiality of *Tb*PMM (Fig. 9A). Phenotypic analyses of this knockdown cell line were carried out at 24 h and 48 h post dsRNA induction. The expression of *Tb*PAGM protein was evaluated by Western blot in case this enzyme, which can interconvert Man-1-P and Man-6-P, might be upregulated in the induced *Tb*PMM RNAi cells to compensate for the loss of *Tb*PMM, but this was not the case (Fig. 9B). Sugar nucleotides were extracted from induced and un-induced *Tb*PAGM and *Tb*PMM RNAi cell lines and from wild type cells, separated by HPLC and quantified by electrospray ionization tandem mass spectrometry, as previously described (Turnock and Ferguson, 2007). In the *Tb*PMM RNAi cell line the GDP-Man levels were reduced upon induction, as expected. Thus, GDP-Man was reduced to 55% and 50% (*P* < 0.005) of wild type levels after 24 and 48 h of dsRNA induction respectively, and the downstream GDP-Man metabolite, GDP-Fuc, was reduced to 40% (*P* = 0.001) of wild type levels after 48 h (Fig. 9C). However, under these latter conditions all of the UDP-sugar levels were similar to those of wild type or un-induced cells (Fig. 9C). These data show that, unlike GDP-Man and GDP-Fuc, the synthesis of UDP-Glc and UDP-Gal is not critically dependent on the expression of *Tb*PMM. Further evidence of mannose starvation in the induced *Tb*PMM RNAi cell line was obtained by Western blotting with anti-VSG antibodies (Fig. 9D). Thus, as already evident after 24 h but very clear after 48 h, *Tb*PMM knockdown leads to under *N*-glycosylation of sVSG221, as previously described for the conditional null mutant of *T. brucei* GDP-Man pyrophosphorylase (Denton *et al*., 2010).

**Fig. 10:**
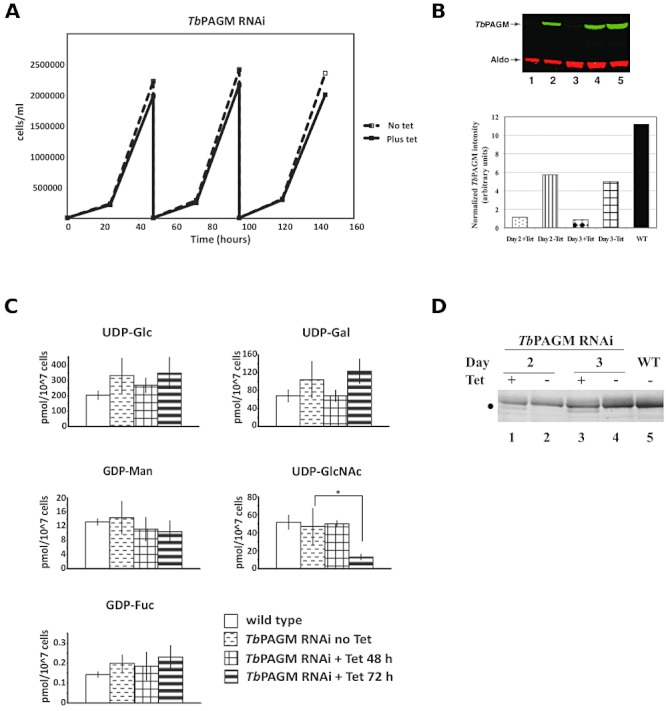
Characterization of *Tb*PAGM RNAi effects. A. Growth curves of the *Tb*PAGM RNAi cell line induced with tetracycline (*solid line*) or un-induced (*dashed line*). B. Western blot for *Tb*PAGM protein in total lysates of wild type cells (lane 5) and *Tb*PAGM RNAi cells un-induced (lanes 2 and 4) or induced (lanes 1 and 3) with tetracycline at day 2 (lanes 1 and 2) and 3 (lanes 3 and 4). The Licor quantitative data, normalized to the aldolase (aldo) signal, are plotted below the blot. C. The levels of sugar nucleotide were determined by LC-MS/MS in wild type cells (*white*), the un-induced *Tb*PAGM RNAi cell line (*stippled*) or the *Tb*PMM RNAi cell line induced for 48 h (*squared*) or 72 h (*bold horizontal stripes*). The *P*-values (*P*) were determined by *t*-test; the asterisk indicates a *P* < 0.05. D. The extent of *N*-glycosylation of sVSG221 was assessed by anti-sVSG221 Western blot of whole cell lysates from wild type (WT) cells (lane 5) and *Tb*PAGM RNAi cells that were induced (+) or un-induced (−) with tetracycline for 2 days or 3 days, as indicated. The lower band in lanes 1 and 3, marked with a spot, is consistent with sVSG221 lacking one of its two *N*-glycans.

The induction of dsRNA targeting *TbPAGM* caused only a slight reduction in cell growth (Fig. 10A), even though the level of *Tb*PAGM protein after 2 and 3 days of induction was reduced to 15% of wild type levels or 30% of un-induced levels respectively (Fig. 10B). No compensatory difference in the RNA levels for *Tb*PMM was observed in the *Tb*PAGM RNAi cell line, either un-induced or induced for 2 or 3 days (data not shown). After 48 h of induction, the sugar nucleotide levels in the *Tb*PAGM RNAi cell line agreed reasonably well with those of un-induced and wild type cells (Fig. 10C). However, after 72 h of induction, the UDP-GlcNAc levels were 25% and 30% (*P* < 0.05) of un-induced and wild type levels respectively, while all other sugar nucleotide levels remained comparable to those in un-induced cells (Fig. 10C). Despite the significant knockdown in *Tb*PAGM protein levels, evidence for GlcNAc starvation, i.e. under *N*-glycosylation of sVSG221 as previously described for the conditional null mutants of *T. brucei* UDP-GlcNAc pyrophosphorylase and glucosamine-6-P *N*-acetyltransferase (Stokes *et al*., 2008; Mariño *et al*., 2011), was barely detectable (Fig. 10D). It was noted previously that growth defects do not occur in bloodstream form *T. brucei* until UDP-GlcNAc levels fall below 20% of wild type levels (Stokes *et al*., 2008) and it would appear that the RNAi knockdown of *Tb*PAGM achieved here is not deep enough to significantly affect protein glycosylation and cell growth.

In conclusion, the analysis of the sugar nucleotide levels in *Tb*PMM and *Tb*PAGM RNAi cell lines suggests that these two enzymes are mutually redundant for the conversion of Glc-6-P to Glc-1-P. Thus, although a reduction in GDP-sugars levels could be observed upon knockdown of *Tb*PMM expression, and likewise reduction in UDP-GlcNAc levels was observed in the case of *Tb*PAGM knockdown, no significant reduction in UDP-Glc or UDP-Gal was observed in either cell line.

## Discussion

Although genes encoding putative PMM and PAGM enzymes were found in the *T. brucei* genome, and biochemically confirmed here, no gene encoding a putative PGM, the enzyme responsible for the synthesis of Glc-1-P from Glc-6-P, could be found (Berriman *et al*., 2005; Turnock and Ferguson, 2007). The absence of a *T. brucei* PGM gene was particularly perplexing. First, because *T. cruzi* and *Leishmania* have easily identifiable *PGM* genes and the *T. cruzi* PGM enzyme has been biochemically characterized (Penha *et al*., 2005; 2009) and, second, because the only known route to the identified *T. brucei* metabolite UDP-Glc (Turnock and Ferguson, 2007) is via UGP (Mariño *et al*., 2010). As UGP uses Glc-1-P and UTP as substrates, and as the only known route to Glc-1-P is from Glc-6-P via PGM activity, it follows that *T. brucei* must possess an enzyme with PGM activity. Furthermore, the flux through UDP-Glc, and therefore also through Glc-1-P, is known to be very considerable in bloodstream form and procyclic form *T. brucei* (Turnock and Ferguson, 2007). This is because UDP-Glc is the obligate precursor of UDP-Gal (Roper *et al*., 2002; 2005; Shaw *et al*., 2003; Urbaniak *et al*., 2006b) that, in turn, is used to synthesize the many galactose-containing glycoproteins of the parasite, including the highly abundant VSG coat glycoprotein and the procyclins in the bloodstream and procyclic forms of the parasite respectively. The biochemical characterization of *Tb*PMM and *Tb*PAGM described in this paper resolves this metabolic paradox, as both enzymes are capable of converting Glc-6-P to Glc-1-P, as well as interconverting their definitive mannose- and N-acetylglucosamine-phosphate substrates. Certain other PMMs and PAGMs have been shown to be similarly promiscuous in other organisms (Fernandez-Sorensen and Carlson, 1971; Boles *et al*., 1994; Hofmann *et al*., 1994; Oesterhelt *et al*., 1996; Pirard *et al*., 1999; Kato *et al*., 2005; Qian *et al*., 2007), but this is the first known example of PGM activity being completely replaced by PMM and/or PAGM. Analysis of the synteny between trypanosomatid genomes (Fig. S7; El-Sayed *et al.*, 2005b) suggests that *T. brucei* has deleted its PGM gene at some point in evolution, presumably after one or both of PMM and PAGM had attained sufficient catalytic flexibility to support Glc-6-P to Glc-1-P conversion as well. The opposite situation was recently reported for another protozoan parasite, *Giardia lamblia*. This organism has no obvious PMM gene but instead has evolved two PGMs: a canonical glucose phosphate-specific PGM and a divergent one able to convert mannose-phosphates (Mitra *et al*., 2010).

The PGM activities of *Tb*PMM and *Tb*PAGM appear to be mutually redundant, such that RNAi knockdown of each in turn has no significant effect on UDP-Glc and UDP-Gal levels. This is despite the fact that Western blot and unpublished proteomic data strongly suggest that *Tb*PAGM is much more abundant than *Tb*PMM. Indeed, it is interesting that *Tb*PAGM, which can also interconvert mannose-phosphates, cannot substitute for *Tb*PMM and that, therefore, RNAi knockdown of *Tb*PMM affects protein glycosylation and suppresses parasite growth. This is consistent with previous studies using conditional null mutants that show that downstream enzymes on the pathway to GDP-Man and its further metabolite GDP-Fuc are essential (Turnock *et al*., 2007; Denton *et al*., 2010). In contrast, the RNAi knockdown of *Tb*PAGM described here was insufficient to significantly affect protein glycosylation or suppress growth. This is despite the fact that the conditional null mutants of glucosamine-6-P *N*-acetyltransferase and UDP-GlcNAc pyrophosphorylase show that the downstream enzymes leading to UDP-GlcNAc are clearly essential (Stokes *et al*., 2008; Mariño *et al*., 2011). The notion that any other phospho-sugar mutase(s) (in addition to *Tb*PMM and *Tb*PAGM) might exist in *T. brucei* that could perform PGM function was excluded by searches of the updated trypanosomatid genomes at TriTrypDB (Aslett *et al*., 2010) for all of the protein family (PFAM) domains relevant to PMM, PAGM and PGM activities (PF03332, PF02878, PF02879, PF02880 and PF00408). These searches returned only PMM and PAGM genes for the African trypanosomes, whereas they returned PMM, PAGM and PGM genes for *T. cruzi* and the Leishmania.

The *Tb*PMM enzyme has some unusual features when compared to other eukaryotic PMMs. First, its apparent *K_m_* for Man-1-P is relatively high (327 ± 66 µM) in comparison to the values reported for *Arabidopsis thaliana* (29.7 µM) (Qian *et al*., 2007), *Galderia sulphuraria* (50 µM) (Oesterhelt *et al*., 1996), and human PMM1 (3.2 µM) and PMM2 (18 µM) (Pirard *et al*., 1997; 1999). Second, unlike the aforementioned enzymes, *Tb*PMM is inhibited by high levels of Man-1-P. Third, whereas PMMs, including that of *L. mexicana* (Kedzierski *et al*., 2006), are typically homodimers, *Tb*PMM appears to be a monomer under physiological conditions. The properties of both *T. brucei* GDP-Man pyrophosphorylase, which shows significant product inhibition (Denton *et al*., 2010), and *Tb*PMM, which shows product inhibition in the Man-6-P to Man-1-P direction, may assist the coupling of GDP-sugar synthesis to glycoprotein synthesis. Thus, a reduction in glycoprotein precursor synthesis would reduce the demand for GDP-Man and lead to an accumulation of Man-6-P, which, via the reversible phosphomannose isomerase reaction, would lead to fructose-6-phosphate that would enter the glycolytic pathway. The ability to divert a significant portion of carbon metabolism into GDP-sugar nucleotides to fuel glycoprotein synthesis in rapidly dividing trypanosomes, and to automatically shunt this back into glycolysis when glycoprotein synthesis is repressed in non-dividing cells, suggests a mechanism that might help trypanosomes manage transformations between non-dividing metacyclic trypomastigotes and rapidly dividing slender trypomastigotes and from slender to non-dividing stumpy trypomastigotes.

In most organisms, PMM and PAGM activities are located in the cytosol. However, in bloodstream form *T. brucei, Tb*PMM localizes to the glycosomes and *Tb*PAGM localizes to both the glycosomes and the cytosol. The glycosomes of *T. brucei* and related trypanosomatids belong, together with the glyoxysomes of plants and peroxisomes of other eukaryotes, to the microbody family of organelles. When compared to the other microbodies, trypanosomatid glycosomes are unique in their essential role in carbohydrate metabolism, i.e. the first seven steps of glycolysis, gluconeogenesis, glycerol metabolism and the pentose-phosphate pathway (Michels *et al*., 2006; Haanstra *et al*., 2008). Glycosomal proteins are typically targeted to these organelles via Peroxisomal Targeting Sequences (PTS), which can be located in the C-terminus (PTS-1) or N-terminus (PTS-2) of a protein. Internal targeting sequences have also been proposed as another targeting mechanism, as in *T. cruzi* PGM and *T. brucei* triosephosphate isomerase (Penha *et al*., 2009; Galland *et al*., 2010) or proteins may reach these organelles by ‘piggybacking’ on other glycosome-targeted proteins (Titorenko *et al*., 2002). As canonical trypanosome PTS sequences are not apparent for *Tb*PAGM or *Tb*PMM [although the *T. cruzi* and *L. major* PMM orthologues do contain PTS-1 sequences (Opperdoes and Szikora, 2006)], it seems likely that both either use the piggybacking mechanism or some unidentified internal targeting sequence. An inefficient import of *Tb*PAGM in the glycosomes could explain the partial localization of this enzyme in the cytosol of bloodstream form *T. brucei*. In any case, both *Tb*PMM and *Tb*PAGM join the growing list of enzymes of *de novo* sugar nucleotide biosynthesis that have been localized to the glycosomes. These include hexokinase and phosphoglucose isomerase (Visser and Opperdoes, 1980), glucosamine-6-P *N*-acetyltransferase (Mariño *et al*., 2011), *Tb*UAP (Stokes *et al*., 2008), *Tb*UGP (Mariño *et al*., 2010), GalE (Roper *et al*., 2005), phosphomannose isomerase (Colasante *et al*., 2006) and GDP-mannose 4,6-dehydratase (Turnock *et al*., 2007). It is possible that all or most of the sugar nucleotide biosynthesis machinery is localized to the glycosome in *T. brucei* and that the rate of synthesis of these metabolites is controlled, to some degree, by ‘compartmentalization’. Such a mechanism has already been proposed as an alternative to allosteric regulation for glycolysis in *T. brucei* (Haanstra *et al*., 2008).

In summary, we have expressed and characterized the PMM and PAGM enzymes of *T. brucei* and determined their subcellular locations and the high-resolution crystal structure of *Tb*PMM. Further, by analysing their substrate specificities, we have provided a solution for the apparently paradoxical absence of a PGM gene in *T. brucei* by demonstrating that both *Tb*PMM and *Tb*PAGM can interconvert Glc-6-P and Glc-1-P. Selective knockdown of *Tb*PMM and *Tb*PAGM by inducible RNAi and analysis of sugar nucleotide levels suggests that they are mutually redundant for this particular function. It is also clear that the routes to three key sugar-1-phosphates (Man-1-P, GlcNAc-1-P and Glc-1-P) and thence to five key sugar nucleotides (GDP-Man, GDP-Fuc, UDP-GlcNAc, UDP-Glc and UDP-Gal) are dependent on just two enzymes: *Tb*PMM and *Tb*PAGM, each of which could have therapeutic potential.

## Experimental procedures

### DNA isolation and manipulation

Plasmid DNA was purified from *Escherichia coli* (DH5α) using the Qiagen Miniprep or Maxiprep kit as appropriate. Gel extraction was performed using QIAquick kits. Custom oligonucleotides were obtained from the University of Dundee oligonucleotide facility. *T. brucei* genomic DNA was isolated from 1 × 10^8^ bloodstream form cells using DNAzol (Helena Biosciences). All *T. brucei* cell cultures are mycoplasma-free.

All plasmids were verified by sequencing (DNA Sequencing Service, College of Life Sciences, University of Dundee; http://www.dnaseq.co.uk). Recombinant proteins were identified in the Proteomics and Mass Spectrometry Facility, College of Life Sciences, University of Dundee.

Protein sequence multiple alignments were assembled using ClustalW (Chenna *et al*., 2003) and Jalview (Waterhouse *et al*., 2009).

### *Tb*PMM expression and purification for antibody production

The *TbPMM* (**Tb927.10.6440**) ORF was amplified by PCR from *T. brucei* genomic DNA with Platinum Taq DNA polymerase High Fidelity (Invitrogen) using forward and reverse primers containing *Nde*I and *Xho*I restriction sites (underlined) respectively: 5′-GGAATTCCATATGAAAAGAGTTCTTTTACTCTTTGAC-3′ and 5′-CCGCTCGAGTTACTTCATGGCAATTATTTTTTCC-3′. The *Nde*I and *Xho*I restriction sites were used to clone the *TbPMM* ORF into a pET15b (Novagen) modified with a PreScission™ Protease (PP) cleavage site, generating the following expression construct: pET15b-His_6_-PP-*TbPMM*. The recombinant *Tb*PMM was expressed in *E. coli* BL21(DE3). The cells were grown overnight at 16°C after induction with 0.75 mM isopropyl β-D-1-thiogalactopyranoside (IPTG). After harvesting, the cells were resuspended in 50 mM Tris-HCl pH 7.3, 150 mM NaCl, 100 mM imidazole, 1 mM DTT, 1 mg ml^−1^ lysozyme and Complete Protease Inhibitor Cocktail Tablets (Roche) and then lysed using a French press. The cell lysate was cleared by centrifugation (17 000 *g*, 4°C, 30 min), filtered through a 0.2 µm cellulose acetate membrane (Whatman) and loaded onto a 5 ml Ni^2+^ HiTrap™ Chelating HP column (GE Healthcare). Fractions were collected and checked by SDS-PAGE. The *Tb*PMM containing fractions were pooled and digested with 0.1 mg of PreScission™ protease GST-tagged (a kind gift of Daan van Aalten, University of Dundee) overnight at 4°C. The PreScission™ protease was removed by incubating the sample for 2 h at 4°C with 50 µl of Glutathione Sepharose™ 4 Fast Flow (GE Healthcare) slurry and then by separating the beads by centrifugation. A fraction of the untagged *Tb*PMM, present in the supernatant, was used for mouse and rabbit immunization. The remaining recombinant protein was used for coupling to CNBr Sepharose beads.

### *Tb*PMM protein expression and purification for activity assays and crystallization trials

*Tb*PMM ORF was amplified as described above and cloned into the pET15-MHL vector (GenBank ID EF456738) and transformed into *E. coli* BL21-(DE3)-V2R-pRARE2. A single colony was inoculated into 100 ml of LB medium containing ampicillin/chloramphenicol (100 µg ml^−1^ and 34 µg ml^−1^ respectively) inside a 250 ml baffled flask and incubated with shaking at 250 r.p.m. overnight at 37°C. The culture was transferred into 1.0 l of TB with the same antibiotic formulation pre-added. The culture was allowed to grow in the LEX system (Harbinger Biotechnology and Engineering) to an OD_600_ of 5–6, cooled to 15°C and induced with 0.5 mM IPTG overnight at that temperature. The culture was harvested by centrifugation. The resulting pellets were resuspended to approximately 40 ml l^−1^ cell culture in a Binding Buffer (50 mM HEPES pH 7.5, 500 mM NaCl, 5 mM imidazole and 5% glycerol) with protease inhibitors (1 mM benzamidine and 1 mM phenylmethyl sulphonyl fluoride) added, and stored at −80°C.

On the day before purification, the pellets were thawed overnight at 4°C. Each pellet from 1 l of culture was pretreated with 0.5% CHAPS and 500 units of benzonase for 40 min at room temperature, and subsequently sonicated. After 6 min sonication, the cell lysate was centrifuged using a Beckman JA-25 rotor at 24 000 r.p.m. for 20 min at 4°C. The cleared lysate was loaded onto a 1.0–2.5 ml Ni-NTA (Qiagen) open column (pre-equilibrated with Binding Buffer) at approximately 1.5–2.0 ml min^−1^. The Ni-NTA column was then washed with 150 ml of Wash Buffer (50 mM HEPES pH 7.5, 500 mM NaCl, 30 mM imidazole and 5% glycerol) at 2–2.5 ml min^−1^. The protein was then eluted with Elution Buffer (50 mM HEPES pH 7.5, 500 mM NaCl, 250 mM imidazole and 5% glycerol). The eluted sample was applied to a Sephadex S200 16/60 gel filtration column (GE Healthcare) pre-equilibrated with Gel filtration Buffer (10 mM HEPES, pH 7.5, 500 mM NaCl) on an AKTA explorer system (GE Healthcare). The fractions corresponding to the eluted protein peak were pooled and further treated with TEV protease overnight to cut the His tag. The mixture was loaded onto another 1.0 ml Ni-NTA open column and the cut protein was collected from the flow through. Its identity and purity were evaluated by mass spectroscopy and SDS-PAGE gel. The sample was then concentrated using a 15 ml Amicon Ultra centrifugal filter device (Millipore) to 10 mg ml^−1^ and stored at 4°C.

### *Tb*PAGM protein expression and purification for activity assays and antibody production

*TbPAGM* (**Tb927.8.980**) was amplified by PCR from *T. brucei* genomic DNA with Platinum Taq DNA polymerase High Fidelity (Invitrogen) using forward and reverse primers containing *Nde*I and *Xho*I restriction sites (underlined) respectively: 5′-GGAATTCCATATGGTGCTGCAGGCT-3′ and 5′-CGCCTCGAGCTACGCTCCACCGCAGA-3′. The *Nde*I and *Xho*I restriction sites were used to clone the *TbPAGM* ORF into a pET15b (Novagen) modified with a PreScission™ Protease (PP) cleavage site, generating the following expression construct: pET15b-His_6_-PP-*TbPAGM*. The recombinant *Tb*PAGM was expressed in *E. coli* BL21(DE3). Cultures were grown at 37°C, in LB medium containing ampicillin, until OD_600_ was approximately 0.5. IPTG was added to a final concentration of 0.5 mM and the cultures were grown overnight at 16°C. The cell pellets were obtained by centrifugation and resuspended in 50 mM Tris-HCl pH 7.7, 200 mM NaCl, 50 mM imidazole, in the presence of DNase I, EDTA-free Complete Protease Inhibitor Cocktail Tablets (Roche) and 1 mg ml^−1^ lysozyme. The cells were lysed using a French press and the cell lysate cleared by centrifugation (20 000 *g*, 4°C, 30 min) filtered through a 0.2 µm cellulose acetate membrane (Whatman) and loaded onto a 5 ml Ni^2+^ HiTrap™ Chelating HP column (GE Healthcare). The *Tb*PAGM-containing fractions were pooled and digested with 0.1 mg of PreScission™ protease GST-tagged as described above. The cleaved protein was further purified by gel filtration using a Superdex G-200 10/30 column (Amersham) and eluted in 50 mM Tris-HCl pH 7.7, 200 mM NaCl.

### Analytical ultracentrifugation

Recombinant and untagged *Tb*PMM was analysed by sedimentation velocity on a Beckman Optima XL-1 Analytical Ultracentrifuge with an AN50-Ti rotor at 4°C, 32 000 r.p.m. The quaternary structure of the protein was studied at 0.25, 0.5 and 0.75 mg ml^−1^ in two different buffers: (i) 10 mM HEPES pH 7.5, 150 mM NaCl, (ii) 10 mM Tris-HCl pH 7.5, 1 mM DTT. Absorbance data were collected and analysed using the SEDFIT software (Schuck, 2004). *Tb*PMM was assumed to be a globular protein and its amino acid composition was used to determine its density.

### Activity assay for the conversion of Man-1-P to Man-6-P

To assay activity, 10 ng of recombinant untagged *Tb*PMM was incubated in 100 µl reaction volume for 20 min at room temperature in buffer D (2 mM Bis-Tris propane pH 7.3, 5 mM MgCl_2_, 1 µM glucose-1,6-biphosphate, 50 ng of BSA and 250 µM Man-1-P). For *Tb*PAGM, 100 ng of enzyme was incubated in buffer D for 1 h at 37°C. The reaction was then stopped by addition of an equal volume of 0.2 M NaOH, raising the pH above 12. The samples were analysed by High Pressure Anion Exchange Chromatography coupled to a Pulse Amperometric Detector (HPAEC-PAD, Dionex) using a CarboPac PA1 column and conditions adapted from Zhou *et al*. (2002). For the kinetic analysis, the concentration of Man-1-P in the reaction was varied between 10 and 1000 µM. Each concentration was analysed in triplicates. A high-substrate inhibition equation (1) based on a non-linear fit was used to calculate the kinetic parameters of the reaction.


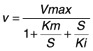
(1)

All kinetic data were fitted using GraFit5.

### Activity assays for the conversion of Glc-6-P and Glc-1-P

The conversion of Glc-1-P to Glc-6-P by recombinant *Tb*PMM and *Tb*PAGM was first followed by HPAEC-PAD. The conditions and reaction buffer are the same as described above for the Man-1-P to Man-6-P conversion, except that 25 ng of enzyme was used and the substrate was changed to Glc-1-P.

The conversion of Glc-6-P to Glc-1-P by *Tb*PMM and *Tb*PAGM was also analysed using a coupled assay with *Tb*UGP. The formation of the final product (UDP-glucose) was followed on a HPAEC system (Dionex) using a CarboPac PA1 column and a UV detector set at 260 nm. Different amounts of recombinant proteins (50–400 ng) were incubated 15 min at room temperature in 100 µl reaction volume containing 2 mM Bis-Tris propane pH 7.3, 10 mM MgCl_2_, 1 µM glucose-1,6-biphosphate, 500 µM Glc-6-P, 1 mM DTT, 0.1 mg ml^−1^ BSA, 500 µM UTP and 1 µg rx^−1^*Tb*UGP (Buffer CA). The reaction was stopped by addition of an equal volume of 0.2 M NaOH.

Kinetic parameters were determined using a colorimetric assay on a 96-well plate format (Cellstar) as described in Stokes *et al*. (2008). Briefly, 25 ng of recombinant *Tb*PMM or *Tb*PAGM was incubated in 100 µl reaction volume for 15 min at room temperature in buffer CA, containing also 0.08 U rx^−1^ pyrophosphatase (Sigma). The concentration of Glc-6-P was varied between 10 and 1000 µM. After addition of 100 µl of Biomol Green (Biomol International) and 20 min incubation, the A_620_ was measured on a SpectraMax 340PC (Molecular Devices) plate reader. Triplicates were run for each point. The same high-substrate inhibition equation as above was used to fit the kinetic data for *Tb*PAGM.

IC_50_ for GlcNAc-1-P and GlcNAc-6-P was measured using the colorimetric coupled assay. Briefly, 25 ng of *Tb*PAGM was incubated in the same conditions described above. A fixed concentration for Glc-6-P of 100 µM was used, while the GlcNAc-1-P and GlcNAc-6-P concentration was varied between 1 and 250 µM. The detection was carried out as above.

### Activity assays for the conversion of GlcNAc-6-P and GlcNAc-1-P

The conversion of GlcNAc-1-P to GlcNAc-6-P by recombinant *Tb*PAGM was first followed by HPAEC-PAD. The conditions and reaction buffer (Buffer D) are the same as described above for the Man-1-P to Man-6-P conversion, expect that 25 ng of enzyme was used and the substrate was changed to GlcNAc-1-P. For *Tb*PMM, 250 ng of enzyme was incubated in Buffer D containing 800 µM GlcNAc-1-P for 1 h at 37°C before analysis by HPAEC-PAD.

The conversion of GlcNAc-6-P to GlcNAc-1-P by *Tb*PAGM was analysed using a coupled assay with *Tb*UAP with colorimetric detection on a 96-well plate format (Cellstar) as described above. Briefly, 25 ng of recombinant *Tb*PAGM was incubated in buffer CA (2 mM Bis-Tris propane pH 7.3, 10 mM MgCl_2_, 1 µM glucose-1,6-biphosphate, 1 mM DTT, 0.1 mg ml^−1^ BSA, 500 µM UTP and 1.5 µg rx^−1^*Tb*UAP). Kinetic parameters were determined by varying the concentration of GlcNAc-6-P between 5 and 100 µM. Triplicates were run for each point.

### Parasite culture

Bloodstream form *T. brucei* parasites (strain 427, variant MITat1.2) expressing T7 polymerase and tetracycline repressor protein under G418 selection (Wirtz *et al*., 1999) were grown in HMI-9t medium at 37°C with 5% CO_2_. HMI-9T is a variant of the HMI-9 medium described in Hirumi and Hirumi (1994) where thioglycerol is used instead of β-mercaptoethanol (Greig *et al*., 2009).

### Generation of RNAi constructs and transformation of bloodstream form *T. brucei*

RNAit (http://trypanofan.path.cam.ac.uk/software/RNAit.html; Redmond *et al*., 2003) was used to identify suitable RNAi internal coding sequences for *TbPMM* (**Tb927.10.6440**, nt. 204–667) and *TbPAGM* (**Tb927.8.980**, nt. 676–1172). The 464 nt (*TbPMM*) and 497 bp (*TbPAGM*) fragments, containing *Xho*I and *BamH*I restriction sites at their 5′ and 3′ respectively, were synthesized by Dundee Cell Products and were then cloned into p2T7TA^Blue^ (Alibu *et al*., 2005) using the *Xho*I and *BamH*I sites to generate p2T7-*TbPMM* and p2T7-*TbPAGM*. These constructs were digested with NotI, precipitated, washed with 70% ethanol and redissolved in sterile water, before being used for *T. brucei* transformation by Amaxa nucleofection as previously described (Burkard *et al*., 2007). The final constructs were verified by sequencing (DNA Sequencing Service, College of Life Sciences, University of Dundee) before transformation.

### Sugar nucleotide analysis

Sugar nucleotide extraction and analysis were performed as previously described (Turnock and Ferguson, 2007). Briefly, cells were pelleted by centrifugation, washed in ice-cold phosphate buffer saline and lysed in 70% ethanol in the presence of 20 pmol of the GDP-glucose internal standard (Sigma). The lysate was centrifuged to remove insoluble material and the supernatant extracted with butan-1-ol to remove lipids. Sugar nucleotides were extracted from the resulting aqueous phase using EnviCarb graphitized carbon columns (Supelco) as previously described (Rabina *et al*., 2001). The eluted sugar nucleotides were analysed by multiple reaction monitoring LC-MS/MS (Turnock and Ferguson, 2007). The *P*-values were determined using Student's *t*-test.

### Crystallization and data collection

Crystals for *Tb*PMM were obtained by setting up the protein sample obtained as described above in a hanging drop vapour diffusion experiment at 20°C. The successful crystallization conditions leading to the structure were: 2 M (NH_4_)_2_SO_4_, 0.2 M NaCl, 0.1 M sodium cacodylate, pH 6.0.

Crystals for data collection were transferred to paratone-N as a cryoprotectant, flash frozen in liquid nitrogen and data collected on a Rigaku FRE Superbright anode with a RAXIS-IV plate imager. A complete data set was collected, then indexed and scaled with the HKL2000 program (Otwinowski and Minor, 1997).

### Structural determination and model refinement

The structure for *Tb*PMM was solved by molecular replacement using the program PHASER and the *Lmex*PMM structure (**2i54**) modified to have its side-chains replaced with the Tb sequence by FFAS03 (Jaroszewski *et al*., 2005). The model was refined against 1.85A data using an initial round of ARP/WARP, followed by iterative manual rebuilding in COOT and refinement with REFMAC 5.5.0109 (ccp4 Suite, Murshudov *et al*., 1997). The final model was refined with good geometry and statistics, and checked with MOLPROBITY with no outliers in the Ramachandran plot. Final data information can be found in Table 2. *Tb*PMM residues in this paper are numbered from the start methionine.

### Production of antibodies and cell localization of *Tb*PMM and *Tb*PAGM by immunofluorescence

Two adult Balb/c mice were used to raise polyclonal antibodies against *E. coli* overexpressed and untagged *Tb*PMM protein with Freund's complete adjuvant. Each animal received two further immunizations with Freund's incomplete adjuvant over 2 months. A pool of these mice sera was used in Western blots and immunofluorescence. Similar procedures were performed for *Tb*PAGM.

For the immunofluorescence experiments, wild type bloodstream form *T. brucei* cells were grown in HMI-9T medium (Hirumi and Hirumi, 1994; Greig *et al*., 2009) to a density of 1 to 2 × 10^6^ cells ml^−1^, harvested by centrifugation at 800 *g* for 10 min at 4°C and resuspended in trypanosome dilution buffer (20 mM Na_2_HPO_4_, 2 mM NaH_2_PO_4_, 5 mM KCl, 80 mM NaCl, 1 mM MgSO_4_, 20 mM glucose pH 7.8) to a density of 2 × 10^7^ cells ml^−1^. Aliquots (10 µl) were placed on 13 mm pre-cooled coverslips inside 12-well plate, covered, incubated for 15 min at 4°C and subsequently fixed in 1 ml of freshly prepared 4% paraformaldehyde in phosphate-buffered saline (PBS) for 30 min at 4°C followed by three washes with 1 ml of PBS. Cells were permeabilized with 0.1% Triton X-100 in PBS for 10 min at room temperature. Samples were then blocked in 5% fish skin gelatin (FSG) in PBS containing 10% normal goat serum. The coverslips were incubated in a humid chamber with 1:1000 mouse anti-*Tb*PMM mixed with either 1:2000 rabbit anti-glyceraldehyde-3-phosphate dehydrogenase (GAPDH) antiserum or 1:4000 rabbit anti-enolase antiserum diluted in 1% FSG in PBS, 0.05% Triton X-100. Both anti-GAPDH and anti-enolase were kindly provided by Paul Michels (Catholic University of Louvain, Belgium). Samples were subsequently washed with 1% FSG in PBS, 0.05% Triton X-100 and incubated with 50 µl of 1:500 diluted Alexa 488-conjugated goat anti-mouse IgG and 1:500 diluted Alexa 594-conjugated goat anti-rabbit IgG for 1 h. Coverslips were washed and mounted on glass slides over a drop of Hydromount containing 2.5% DABCO and left to set in the dark for 30 min. Microscopy was performed on a Zeiss LSM 510 META confocal microscope.

### Digitonin latency and Western blotting

Digitonin latency was performed as previously reported (Galland *et al*., 2007). Briefly, *T. brucei* bloodstream form cells (2 × 10^9^ cells) were washed twice with 10 ml of STE buffer (250 mM sucrose, 25 mM Tris-HCl pH 7.4 and 1 mM EDTA) and resuspended in 1.5 ml of STEN (STE containing 0.15 M NaCl). Aliquots (0.15 ml) were treated with equal volume of digitonin at various concentrations in the presence of 0.1 mM TLCK, 1 µg ml^−1^ leupeptin, 1 µg ml^−1^ aprotinin and 1 mM PMSF. Digitonin stock was prepared at 10 mg ml^−1^ in DMSO and diluted in STEN to the required concentrations. Complete extraction was obtained in parallel by treating an equivalent number of cells with 0.1% Triton X-100 in STEN. The lysates were incubated for 5 min at room temperature and the insoluble materials removed by centrifugation at 16 000 *g* for 2 min. The pellets were discarded; aliquots of the supernatants were run on a reducing 4–12% gradient NuPage gel (Invitrogen) and transferred to nitrocellulose for Western blotting. Note: Different amounts of same samples were loaded onto separate gels for each antibody Western blot, in order to keep within the detection by ECL and film exposure linear during the Western blot development; for *Tb*PMM 1 × 10^7^ cell equivalents per lane, for *Tb*PAGM 5 × 10^6^ cell equivalents per lane, for aldolase (aldo) 1 × 10^5^ cell equivalents per lane, for PEX13 5 × 10^6^ per lane and for enolase (eno) 1 × 10^5^ cell equivalents per lane. The membranes were blocked with 0.25% bovine serum albumin, 0.05% Tween-20 (Sigma), 0.15 M NaCl in 50 mM Tris-HCl pH 7.4. The membranes were probed in parallel with the following antibodies 1:1000 diluted mouse anti-*Tb*PMM, 1:1000 mouse anti-*Tb*PAGM, 1:4000 rabbit anti-aldolase, 1:5000 rabbit anti-Pex13 or 1:5000 rabbit anti-enolase for 1 h at 37°C. The membranes were washed and incubated for 1 h at room temperature with 1:100 000 goat anti-mouse or goat anti-rabbit horseradish peroxidase conjugate. After washing, the membranes were developed by chemiluminescent detection (Super Signal WestPico, Thermo Scientific) and film (Hyperbond ECL, GE Healthcare).

For the immunoprecipitation of native *Tb*PMM (to check its mono-specificity), 2 × 10^8^*T*. *brucei* bloodstream form cells were lysed in 1% (w/v) SDS in 20 mM Tris-HCl pH 6.8 containing 0.1 M DTT and heated at 50°C for 15 min. SDS was diluted to 0.03% with 1% (w/v) Triton X-100 in 20 mM Tris-HCl pH 6.8 with 0.15 M NaCl in the presence of 0.1 mM TLCK, 1 µg ml^−1^ leupeptin, 1 µg ml^−1^ aprotinin and 1 mM PMSF. The insoluble material was removed by centrifugation, and 5 µl of pooled mice anti-*Tb*PMM sera was added to the supernatant and incubated for 1 h at 4°C. Subsequently, 50 µl of protein G Dynabeads (Invitrogen) was added and incubated for 1 h at 4°C. The beads were recovered by placing on the magnet for 2 min and washed three times with cold 20 mM Tris-HCl pH 7.2, 0.15 M NaCl, 0.03% SDS and 1% Triton X-100. The beads were boiled in SDS sample buffer with 0.1 M DTT, run on a 4–12% Nupage gel and Western blotted as described above.

### Western blot anti-*Tb*PAGM and anti-sVSG

*Tb*PAGM RNAi cells were induced or not with tetracycline for 2 and 3 days, lysed in SDS sample buffer, loaded (5 × 10^6^ cell equivalents per lane) on SDS-PAGE gel and blotted to nitrocellulose. The membrane was blocked with 2% (w/v) FSG in blocking buffer described above and developed with 1:1000 mouse anti-*TbP*AGM mixed with 1:4000 rabbit anti-aldolase. The membrane was washed three times with 0.1% Tween-20 in PBS, incubated with 1:15 000 donkey anti-mouse green infrared conjugate (IRDye 800 CW, Licor) mixed with 1:20 000 donkey anti-rabbit red conjugate (IRDye 680, Licor) and scanned in a Licor Odyssey Infrared Imaging System. The Licor imager software was used to quantify the signal for both infrared channels and the gel loading was normalized against the aldolase signal. For the sVSG analysis, *Tb*PAGM RNAi cells were induced or not with tetracycline for 2 and 3 days while *Tb*PMM RNAi cells for 2 days, then they were lysed in SDS sample buffer, loaded (5 × 10^6^ cell equivalents per lane) on SDS-PAGE gel and Western blotted to nitrocellulose. The Licor development was as described above, but instead rabbit anti-sVSG221 was used at 1:2000 dilution.

### Quantitative reverse transcriptase PCR (qRT-PCR)

RNA was extracted using the RNeasy RNA extraction kit (Quiagen). cDNA was synthesized from 1 µg of RNA using oligo(dT) and random primers with the qScript cDNA synthesis system (Quanta Biosciences), and then diluted 1 in 100 with nuclease-free water. qRT-PCR reactions included 3 µl of diluted cDNA, SYBR Green Master Mix (Quanta Biosciences), and 0.6 µM forward (atgagggctttgataaagcgagc) and reverse (cgtccgagttgctcaacctgc) gene-specific primers. Amplification was carried out in an iCycler iQ5 PCR thermal cycler (Bio-Rad). Mean and standard deviation were determined by combining four replicate analyses.
