# Supplementary material for: Phosphoglucomutase is absent in Trypanosoma brucei and redundantly substituted by phosphomannomutase and phospho-N-acetylglucosamine mutase
Source: Mol Microbiol. 2012 Jul 12;85(3):513–34. doi: 10.1111/j.1365-2958.2012.08124.x (PMC3465800; doi:10.1111/j.1365-2958.2012.08124.x)
Supplement: Supplementary file 1 [file mmi0085-0513-SD1.pdf]

Supporting Information

Phosphoglucomutase is absent in *Trypanosoma brucei* and redundantly substituted by phosphomannomutase and phospho-*N*-acetylglucosamine mutase.

Giulia Bandini[1,2], Karina Mariño[1,3], M. Lucia Sampaio Güther[1], Amy Wernimont[4], Sabine Kuettel[1], Wei Qiu[4], Shamshad Afzal[1,5], Anna Kelner[1], Raymond Hui[4], Michael A. J. Ferguson[1,*].

[1]Division of Biological Chemistry and Drug Discovery, College of Life Sciences, University of Dundee, Dundee, DD1 5EH, United Kingdom.

[4]Structural Genomics Consortium, University of Toronto, Toronto, Ontario, Canada.

[*]Corresponding author. Mailing address: Division of Biological Chemistry and Drug Discovery, College of Life Sciences, University of Dundee, Dow St., Dundee DD1 5EH, Scotland, United Kingdom. Phone: 44-1382-384219. Fax: 44-1382-348896. E-mail: m.a.j.ferguson@dundee.ac.uk

[2]Present address: Department of Molecular and Cell Biology, Boston University, Henry Goldman School of Dental Medicine, Boston MA 02118, USA.
[3]Present address: National Institute for Bioprocessing Research and Training, Dublin-Oxford Glycobiology Laboratory, Conway Institute, University College Dublin, Belfield, Dublin 4, Ireland.
[5]Present address: Division of Gene Regulation and Expression, College of Life Sciences, University of Dundee, Dundee, DD1 5EH, United Kingdom

Supporting Figure Legends

FIGURE S1. Recombinant *Tb*PMM purification for activity assays and crystallization trials. The His_6-tagged *Tb*PMM was overexpressed in *E.coli* and purified by metal chelate affinity chromatography followed by size exclusion chromatography. The tag was removed before the

protein was used for activity assays or crystallization trials. Lane *1*: elution after nickel column chromatography. Lane *2*: concentrated protein after gel filtration. Lane *3*: untagged recombinant *Tb*PMM.

FIGURE S2. Recombinant *Tb*PAGM expression and purification for activity assays. The His_6-tagged *Tb*PAGM was overexpressed in *E.*coli and purified on a metal affinity chromatography column. The tag was removed and the recombinant *Tb*PAGM further purified by size exclusion chromatography. Lane *1*: uninduced total cell protein (UTCP); Lane *2*: induced total cell protein (ITCP); Lane *3*: unsoluble fraction after lysis (pellet); Lane *4*: soluble fraction after lysis (s/n); Lane *5*: flow through from the metal affinity chromatography (FT); Lane *6*: elution from the metal affinity chromatography (E); Lane *7*: *Tb*PAGM after cleavege of the tag and size exclusion chromatography (PAGM).

FIGURE S3. Substrate specificity of *Tb*PMM and *Tb*PAGM. A. Recombinant *Tb*PMM was not able to convert GlcNAc-1-P to GlcNAc-6-P (*black chromatogram*). The HPAED-PAD chromatograms for the sugar-phosphates standards (*black dashed*) and the reaction without *Tb*PMM (*gray*) are also shown. *B*. Recombinant *Tb*PAGM was able to convert Man-1-P to Man-6-P (*black*). The chromatograms for the reaction with no enzyme (*gray*) and the sugar-phosphates standards (*black dashed*) are shown in the same panel.

FIGURE S4. Conversion of Glc-1-P to Glc-6-P by *Tb*PMM and *Tb*PAGM. *Tb*PMM and *Tb*PAGM were incubated in the presence of Glc-1-P and Glc-1,6-biP, as indicated. The products

of the reactions were analyzed by HPAEC with pulse amperometric detection. Formation of Glc-6-P was observed with both *Tb*PMM (panel A) and *Tb*PAGM (panel B). No sugar nucleotide peak could be detected in the absence of either phospho-sugar mutase.

FIGURE S5. Analytical ultracentrifugation of recombinant *Tb*PMM. *Tb*PMM at 0.75 mg/ml was analysed by sedimentation velocity in *A*. 10 mM HEPES pH 7.5, 150 mM NaCl or *B*. 10 mM TrisHCl pH 7.5, 1 mM DTT. In both buffer conditions, *Tb*PMM was a very pure monomer with no signs of aggregation, degradation products or oligomers.

FIGURE S6. *Tb*PMM crystal structure. The asymmetric crystal unit contained two polypeptides, A and B. When the symmetry mates were extended, each monomer formed a dimer with its own symmetry mate – ie A:A and B:B.

FIGURE S7. Chromosomal location of the putative *L. major* and *T. cruzi* PGM genes. The putative *L. major* PGM gene (*black box*) and the characterized *T. cruzi* PGM are found in chromosomal locations with considerable synteny between the there trypanosomatids. However, the *T. brucei* PGM homologue appears to have been deleted sometime after speciation. This image was taken from the genome viewer at TriTryDB.

Supporting Figures

Figure S1

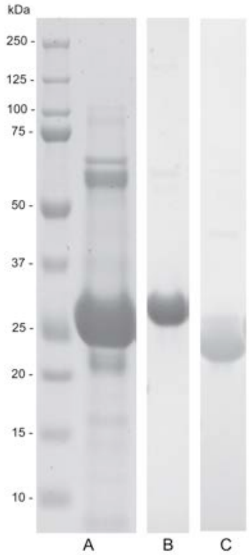

Figure S2

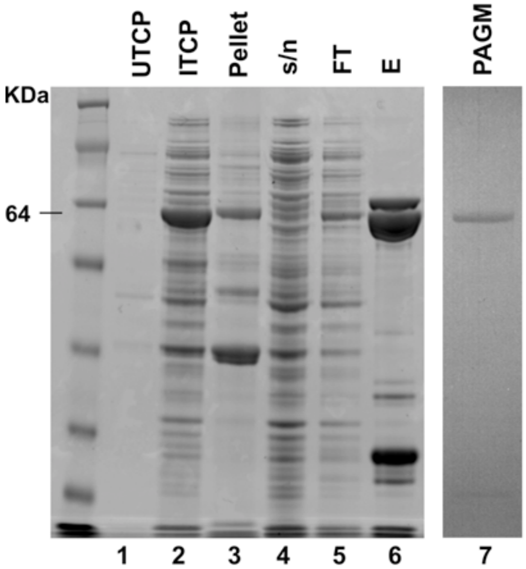

Figure S3

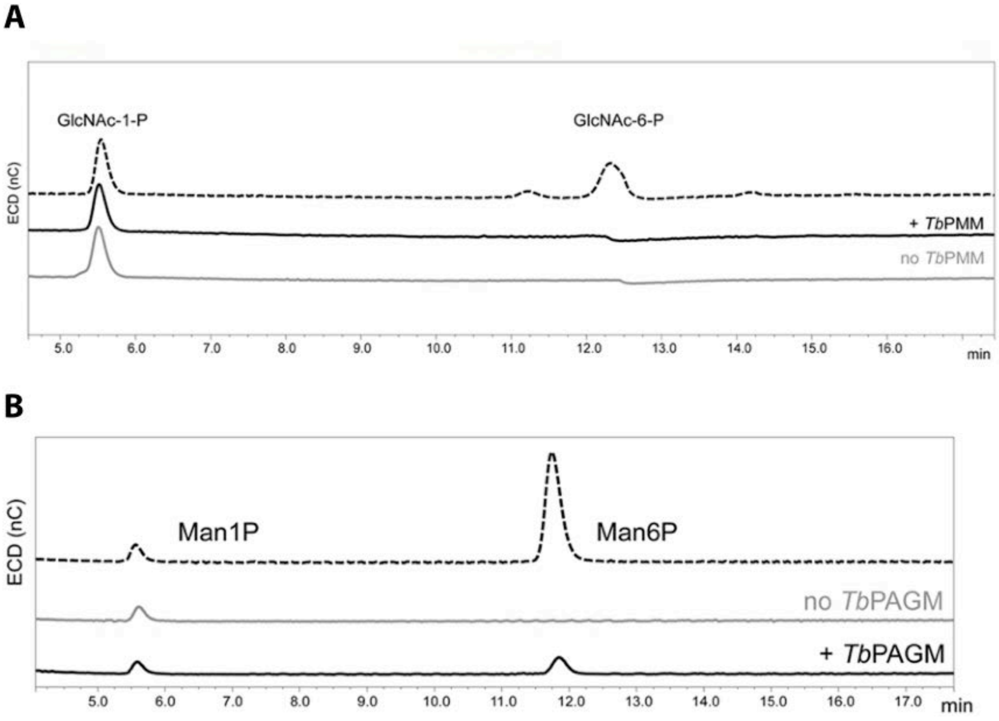
A
GlcNAc-1-P
GlcNAc-6-P
ECD (nC)
+ *Tb*PMM
no *Tb*PMM
5.0 6.0 7.0 8.0 9.0 10.0 11.0 12.0 13.0 14.0 15.0 16.0 min
B
Man1P
Man6P
ECD (nC)
no *Tb*PAGM
+ *Tb*PAGM
5.0 6.0 7.0 8.0 9.0 10.0 11.0 12.0 13.0 14.0 15.0 16.0 17.0 min

Figure S4

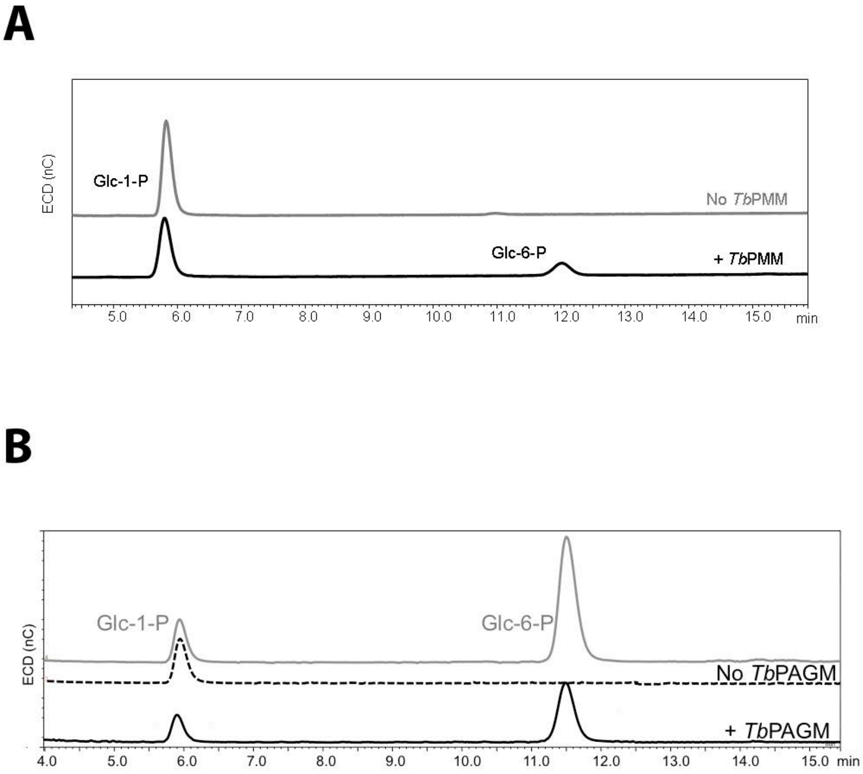

A
ECD (nC)
Glc-1-P
No TbPMM
Glc-6-P
+ TbPMM
5.0
6.0
7.0
8.0
9.0
10.0
11.0
12.0
13.0
14.0
15.0
min
B
ECD (nC)
Glc-1-P
Glc-6-P
No TbPAGM
+ TbPAGM
4.0
5.0
6.0
7.0
8.0
9.0
10.0
11.0
12.0
13.0
14.0
15.0
min

Figure S5

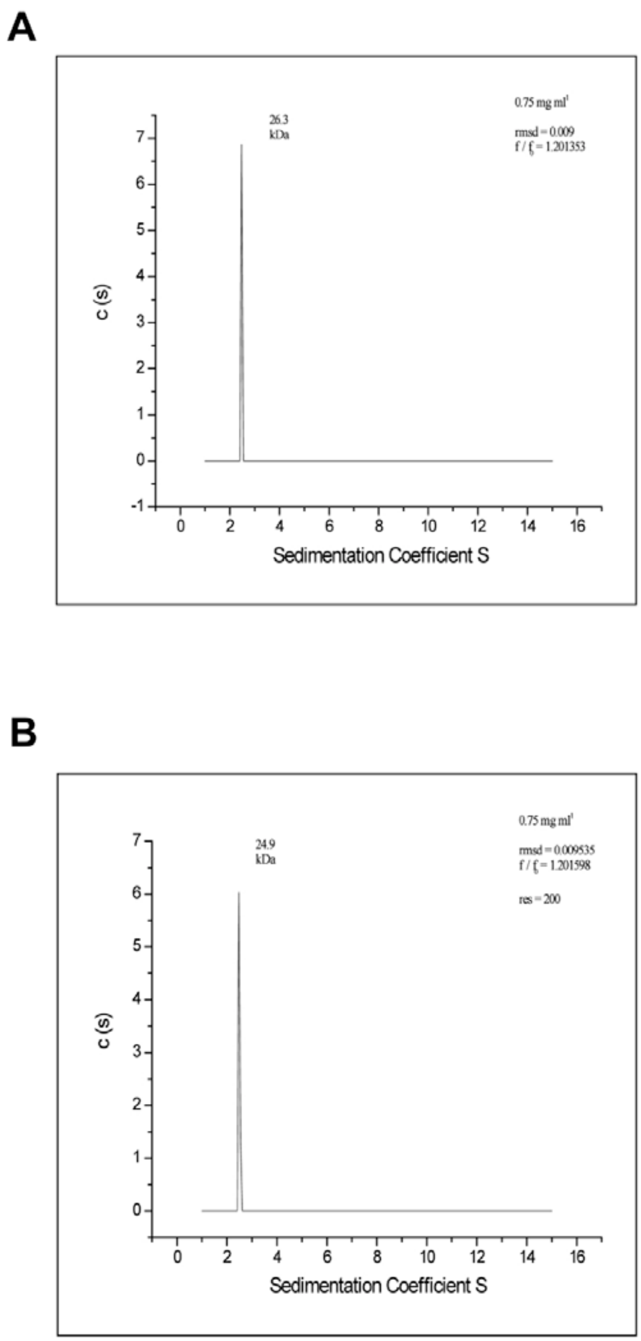
A
0.75 mg ml-1
26.3
kDa
rmsd = 0.009
f / f0 = 1.201353
c (s)
Sedimentation Coefficient S
B
0.75 mg ml-1
24.9
kDa
rmsd = 0.009535
f / f0 = 1.201598
res = 200
c (s)
Sedimentation Coefficient S

Figure S6

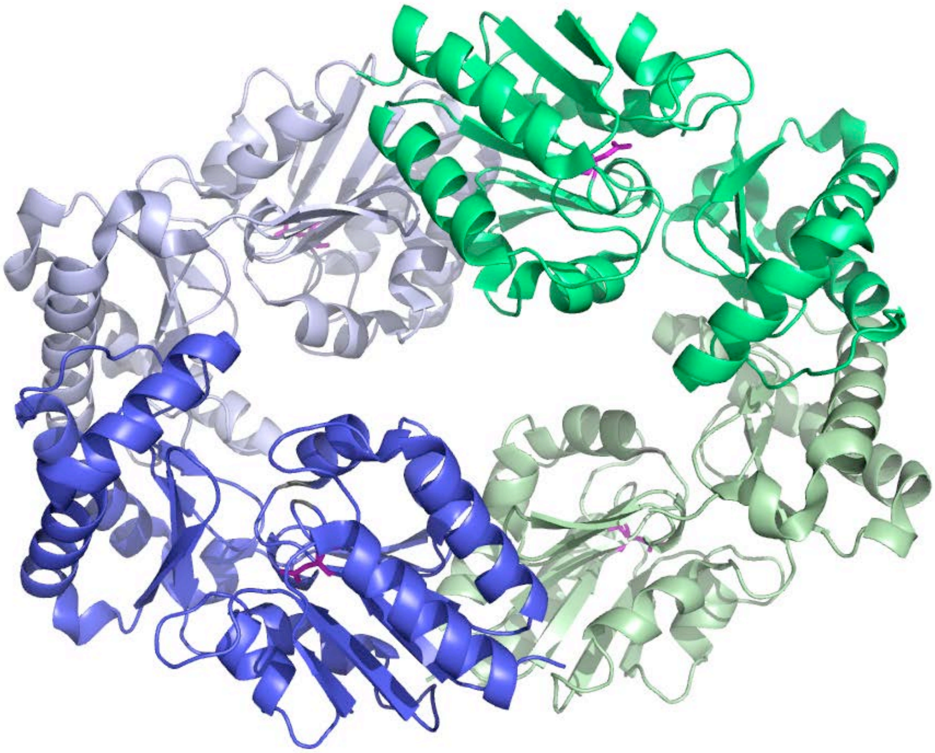

Figure S7

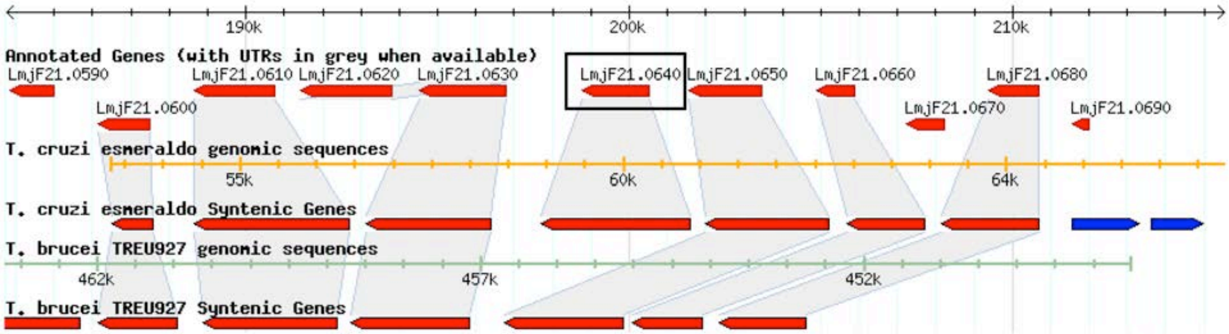
190k
200k
210k
Annotated Genes (with UTRs in grey when available)
LmjF21.0590
LmjF21.0610
LmjF21.0620
LmjF21.0630
LmjF21.0640
LmjF21.0650
LmjF21.0660
LmjF21.0680
LmjF21.0600
LmjF21.0670
LmjF21.0690
T. cruzi esmeraldo genomic sequences
55k
60k
64k
T. cruzi esmeraldo Syntenic Genes
T. brucei TREU927 genomic sequences
462k
457k
452k
T. brucei TREU927 Syntenic Genes
